# How are the mealtime experiences of people in residential aged care facilities informed by policy and best practice guidelines? A scoping review

**DOI:** 10.1186/s12877-022-03340-9

**Published:** 2022-09-09

**Authors:** Rui Ting Grace Koh, Abirami Thirumanickam, Stacie Attrill

**Affiliations:** 1 Flinders University, Adelaide, Australia; 2 School of Allied Health Science and Practice, University of Adelaide, Frome Road, Adelaide, SA 5000 Australia

**Keywords:** Aged care, Long term care, Mealtime, Policy, Structuration theory, Residential care

## Abstract

**Background:**

Mealtimes are embedded routines of residents living in residential aged care facilities (RACFs) that directly impact their health and quality of life. Little is known about how mealtime experiences are informed and affected by structures such as government and organisational policies and processes. This scoping review used Giddens’ (The constitution of society: outline of the theory of structuration, 1984) Structuration Theory to investigate how governance structures related to mealtime practices inform residents’ mealtime experiences.

**Methods:**

Using Arksey and O’Malley’s (Int J Soc Res Methodol 8:19–32, 2005) scoping review framework, a systematic database, grey literature and policy search was completed in May 2020 and updated in July 2021. From 2725 identified articles, 137 articles were included in data charting and deductive analysis, and 76 additional Australian government policy papers were used interpretatively.

**Results:**

Data charting identified that the included studies were prominently situated in Western countries, with a progressive increase in publication rate over the past two decades. Qualitative findings captured structures that guide RACF mealtimes, how these relate to person-centred mealtime practices, and how these facilitate residents to enact choice and control.

**Conclusions:**

Current policies lack specificity to inform the specific structures and practices of RACF mealtimes. Staff, residents, organisational and governance representatives possess different signification, legitimation and domination structures, and lack a shared understanding of policy, and how this influences processes and practices that comprise mealtimes.

**Supplementary Information:**

The online version contains supplementary material available at 10.1186/s12877-022-03340-9.

## Introduction

There are several benefits of positive mealtime experiences on quality of life (QoL) and overall health for people who reside in residential aged care facilities (RACFs) [1]. However, mealtimes are highly variable depending on local RACF practices [2, 3], and are also informed by broader government regulatory processes, local policies and guidelines [4]. Mealtime interventions designed to improve resident nutrition and/or mealtime enjoyment are often implemented in a ‘bottom-up’ format by staff, families or the residents themselves [5]. However, the outcomes of these interventions are variable and often lack generalisation. Whilst these ‘bottom-up’ change processes are often driven by the individuals most affected by mealtime practices, this lacks the ‘top-down’ endorsement of RACF management who have more power and resources to affect meaningful change, or government and local policies and guidelines that may influence mealtime experiences. However, little is understood about the nature of this influence, nor how these governance structures could inform future mealtime interventions.

For residents, their histories, meanings and memories of food frame their understanding of mealtimes [6]. As mealtimes are among the most time-consuming of daily activities, residents’ perceptions of QoL are inevitably linked with eating, nutrition and meals [7]. Speroff and colleagues ([8] p1) suggested that mealtimes in RACFs should “foster independence, promote self-esteem, and make the resident as comfortable and safe as possible, while providing a nourishing, pleasant meal and minimising negative health outcomes”.

Positive mealtimes are associated with quality care, relating to the number of qualified nursing staff in the organisation, the requirements of unlicenced personal carers to complete specialised practices, for example with residents with dementia or palliative care needs, and the degree of carer workload and stress [9–11]. Low staffing reduces time spent with residents, and fosters resident neglect when feeding, unsafe feeding practices, increased choking risk and reduced mealtime social interaction [2]. Positive staff-resident interactions increase resident food consumption and improve mealtime experiences [12]. There is also growing evidence that communal dining methods, such as restaurant-style or family-style dining, create positive mealtime experiences through fostering choice of tablemates and social interaction [13–15]. Similarly, dining room ambience may be modified to promote homeliness [16]. A positive dining culture that fosters social relationships amongst residents enables social normalcy, a sense of belonging and increases interaction opportunities [4, 6, 16]. Conversely, resident factors, including personality differences, health conditions or difficulties complying with RACF routines generate negative mealtime experiences and social anxiety [17]. For example, health conditions that affect a resident’s eating or swallowing function may require texturemodified diets (TMDs) to reduce aspiration and choking risk, but these are known to negatively affect meal enjoyment, calorie intake and QoL [18–21]. Thus, social and physical mealtime environments influence nutrition outcomes and resident QoL [4, 13, 22]. These factors are influenced by government policy and organisational practices that guide how RACFs resource and design mealtimes [23], but it is unclear how these mealtime quality indicators are measured.

Despite evidence demonstrating the benefit for residents of mealtime environments that encourage choice and independence, logistical barriers often prevent RACFs from adopting these approaches [24]. Person-centred care (PCC) approaches provide choice and control on when, what and where to eat, encouraging mealtime participation that improves resident QoL, and are considered best-practice [12, 23, 25]. While some facilities attempt to facilitate person-centred mealtimes, dining practices remain largely staff-directed, staff-enhanced dependency is a common RACF phenomenon, and residents are often excluded from decisions or opportunities to exercise autonomy [8, 24, 26]. Resident participation in decision-making and enactment of mealtimes is thus limited by the RACF’s care approach and practices.

Government policies and standards that regulate and fund RACFs influence local RACF practices and processes [9, 27, 28]. However, it is not known how these government level structures influence how RACFs enact mealtimes, their capacity to implement best person-centred mealtime practices, nor how mealtime quality is conceptualised, measured and assured. Whilst many studies have identified the need for policy that more explicitly addresses RACF mealtime service provision and outcomes [22, 23, 29, 30], it is not clear how policy, practice guidelines or the evidence that informs this influences residents’ mealtime experiences. This scoping review thus aims to explore how evidence about RACF mealtime experiences relates to policy and best practice guidelines. In this study, we have applied the Australian policy context as a case study to explore the broader context of international literature about RACF mealtime experiences. In Australia, most RACFs are private organisations using for-profit or not-for-profit business models, and a limited number of facilities are funded by state governments. However, the Australian federal government contributes individual resident funding and governs RACF regulatory procedures that direct safety and quality standards that facilities must adhere to in order to retain their accreditation and funding to operate [31–33]. We consider policy and guidelines as structures that influence RACF mealtimes, and explore these using Gidden’s (1984) Structuration Theory, which conceptualises the creation and reproduction of social systems [34, 35]. This scoping review explores the research question:

- How are the mealtime experiences of residents in RACFs informed by policy and best practice guidelines?

## Methods

### Theoretical framework

Elements of Giddens (1984) Structuration theory were used as a deductive, analytic framework to explore how structures inform the actions of stakeholders within the RACF mealtime system, including those of residents, staff and organisational representatives [35]. These structures and actions have dual influence: mealtime structures influence stakeholder actions, and simultaneously, stakeholder actions influence mealtime structures, with both shaping the mealtime experience. Structures provide rules, routines, traditions and resources that guide how individuals operate during mealtimes. Individuals act within these established mealtime structures, but can also influence or change these structures through their actions. Individuals store structures as memory traces that determine how they act during mealtimes, and how they predict the likely outcomes of these actions as mealtimes become repeated, routinised and internalised. The three memory traces, that are referred to as ‘domains’ in this research, are signification, legitimation and domination. In the context of RACF mealtimes, signification refers to how mealtime actions are encoded in language to form meaning. Legitimation refers to how an individual’s actions operate within mealtime norms and routines. Lastly, domination refers to the resources that individuals use to direct power to accomplish mealtime actions. Together, these domains generated a coding framework to explore RACF mealtime experiences [34].

### Search strategy

The Population, Concept, Context protocol [36] was applied to generate terms to formulate the research question and scoping search, which is demonstrated in Additional file 1. The scoping review was undertaken in May 2020 following Arksey and O′Malley’s [37] methodological framework. A subsequent secondary search that used the same strategy was conducted in July 2021.

A systematic search strategy, demonstrated in Additional file 2, was conducted with input from a medical librarian. Nine databases were searched, and Haddaway et al.’s [38] guidelines were applied, using Google Scholar to search the grey literature. Included articles were publications in English. No date limitations were applied to capture if and how RACF mealtime practices have changed over time, as governance and care structures have evolved. Review studies, including systematic, scoping and literature reviews were included, as their respective synthesis and interpretation of the RACF mealtime phenomena was considered important for the deductive qualitative analysis in the current study. An initial search on how policy and best practice inform mealtimes yielded few results. Therefore, two separate database and grey literature searches were completed in May 2020, and repeated in September 2021 – Search A: mealtime experiences in RACFs and Search B: policies and guidelines that relate to mealtimes in RACFs. As policy documents that specifically addressed mealtimes were not identified in Search B, two separate post-hoc hand searches for Australian aged care policy were applied using Google, to facilitate the drilling down to mealtime practices: 1) Australian aged care policies and 2) Australian Royal Commission into Aged Care Quality and Safety. Since policy and regulation varies between countries, the Australian context was chosen as a case study to illustrate policy impacts on RACF mealtimes.

Final search results were imported into EndNote X9 and screened to remove duplicates [39]. The first 200 title results from the grey literature searches were imported, and added to the primary search dataset, shown in Additional file 3 [38].

### Study selection

**Fig. 1:**
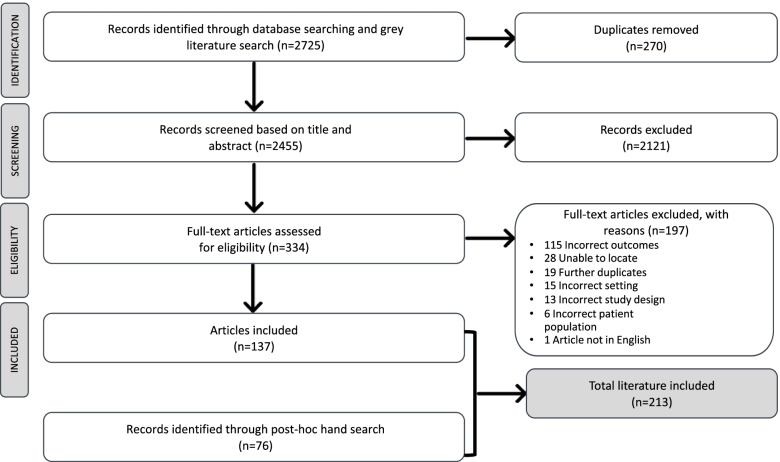
PRISMA flow diagram of included articles

From the primary search (May 2020), 2688 articles were identified and imported into Covidence to manage the screening and full text reviews [40]. Following removal of duplicates, title and abstract screening were completed on 2422 articles. Next, 303 articles were randomly selected for screening by the third author to establish agreement. This yielded a high level of agreement between the researchers (91% and Cohen’s Kappa score of 0.7) [41]. The 34 articles that the authors did not agree on were screened and discussed against the inclusion criteria until a consensus was reached [42]. Figure 1 shows a PRISMA diagram demonstrating the screening process for both the primary and secondary searches [43]. For the primary search the first and third authors completed a full-text review on 319 articles, and 126 of these articles were included for analysis. The secondary search was conducted by the second and third authors, who completed abstract screening on 33 additional articles and full text review on 15 of these, with 11 included for analysis.

The first author compiled memos throughout the primary review process to gather preliminary ideas and identify early categories and themes in the data [44]. Policy documents provided governance structures to interpret the primary data. The original policy search yielded 51 policy documents, with a secondary search, conducted in September 2021 producing a further 25 documents, primarily related to the publication of the final report of the Australian Royal Commission into Aged Care Quality and Safety [45]. Thus, a total of 76 policy documents, grounded within the Australian aged care regulatory context, were used to interpret the scoping review findings.

### Charting the data

Data extraction was completed on the 137 included articles by two researchers independently via Covidence, to obtain quantitative data that was charted against country of research, publication year, methodology and setting in Tables 1, 2 and 3. Extracted data was tabulated, and qualitative analysis was subsequently conducted. NVivo version 12 was used to chart and manage the data [46]. Structure and agency related to mealtime practice was coded according to a deductive framework derived from the three memory trace domains [34]. An inductive qualitative analysis was not applied because the themes captured through this approach were already well represented in the literature.

**Table 1:** Countries of study

Countries	Number of studies	Citations
USA	39	Adams 2013 [47]; Alibrio 1991 [48]; Andreoli 2007 [49]; Aziz and Campbell-Taylor 1999 [50]; Bellomy 2014 [51]; Benedict 1975 [52]; Bertrand 2011 [53]; Bowers 2014 [54]; Buelow and Fee 2000 [55]; Castellanos 2004 [56]; Cohen-Mansfield 1995 [57]; Crogan 2001 [58]; Crogan and Evans 2001 [59]; Dimant 2001 [60]; Dorner 2010 [61]; Escott-Stump 2000 [62]; Evans 2005 [63]; Evans 2004 [64]; Evans and Crogan 2005 [12]; Evans 2003 [65]; Gibson and Barsade 2003 [66]; Hotaling 1990 [67]; Kayser-Jones and Schell 1997a [10]; Kayser-Jones and Schell 1997b [68]; Mahadevan 2014 [69]; McDonnell 2010 [70]; Mikula and Vanaman 2008 [71]; Minniear 1993; Phillips and Van Ort 1995 [72]; Remsburg 2004 [73]; Roberts 2011 [74]; Schell and Kayser-Jones 1999 [75]; Shune and Linville 2019 [30]; Sikorska-Simmons 2007 [76]; Simmons and Levy-Storms 2006 [77]; Simmons 2007 [78]; Simmons 2007 [78]; Simon 2015 [79]; Wu and Barker 2008 [80]
Australia	29	
Canada	21	Chaudhury 2017a [81]; Chaudhury 2017b [82]; Carrier 2009 [1]; Caspar 2020 [83]; Gibbs-Ward and Keller 2005 [84]; Gilbart 2005 [85]; Henkusens 2014 [86]; Hung and Chaudhury 2011 [87]; Hung 2016 [88]; Keller 2017 [19, 29]; Lengyel 2004 [89]; Perivolaris 2006 [90]; Steele 1997 [91]; Way 2011 [92]; West 2003 [93]; Wu 2018 [94]; Wu 2015 [95]; Keller 2021 [96]; Caspar 2021 [97]; Trinca 2021 [98]; Morrison-Koechl 2021 [99]
Mixed	16	Anderson 2016 [100]; Aselage 2011 [7]; Chaudhury 2013 [101]; Fetherstonhaugh 2019 [102]; Hines 2010 [20]; Kayser-Jones 1982 [28]; Keller 2015 [14]; Keller 2014 [103]; Lowndes 2018 [2]; Morris 2018 [104]; Reimer and Keller 2009 [23]; Vucea 2014 [105]; Wang 2018 [106]; Watkins 2017a [6]; Williams 2012 [107]; Ballesteros-Pomar 2020 [21]
United Kingdom	12	Bamford 2012 [108]; Barnes 2013 [13]; Holmes 2019 [109]; Jones 2019 [110]; Murphy 2017 [111]; Philpin 2014 [15]; Stone 2014 [112]; Ullman 2009 [113]; Watkins 2017b [17]; Watkins 2018 [114]; Watkins 2019 [115]; Maluf 2020 [24]
European Union	12	Baur and Abma 2012 [116]; Bungaard 2005; Fjellstrom 2017 [117]; Harnett 2010 [118]; Harnett and Jönson 2017 [119]; Gastmans 1998 [120]; Grøndahl and Aagaard 2016 [25]; Josefsson 2017 [121]; Palacios-Cena 2013 [122]; Palese 2018 [26]; Sydner and Fjellström 2005 [123]; De Wit 2020 [124]
New Zealand	4	Chisholm 2011 [125]; Miles 2019 [126]; Miles 2020 [127]; Nell 2016 [128]
Taiwan	1	Chang and Roberts 2008 [129]
Japan	1	Annear 2016 [130]
Korea	1	Park 2021
**Total**	**136**

**Table 2:** Study methods and methodologies

Design	Number of studies	Citations
Cross-sectional Study	55	Adams 2013 [47]; Bailey 2017 [18]; Beattie 2014 [131]; Bernoth 2014 [132]; Bundgaard 2005 [4]; Chang and Roberts 2008 [129]; Chou 2002 [133]; Chisholm 2011 [125]; Cohen-Mansfield 1995 [57]; Crogan and Evans 2001 [59]; Evans 2004 [64]; Evans 2005 [63]; Evans 2003 [65]; Grøndahl and Aagaard 2016 [25]; Harnett 2010 [118]; Harnett and Jönson 2017 [119]; Hogden 2017 [27]; Holmes 2019 [109]; Hung and Chaudhury, 2011 [87]; Josefsson 2017 [121]; Kayser-Jones and Schell 1997a [10]; Kayser-Jones and Schell 1997b [68]; Kayser-Jones 1982 [28]; Keller 2014 [103]; Lea 2017 [134]; Lengyel 2004 [89]; Mahadevan 2014 [69]; Milte 2018a [135]; Milte 2018b [136]; Milte 2017 [137]; Morris 2018 [104]; Murphy 2017 [111]; Nell 2016 [128]; Palacios-Cena 2013 [122]; Palese 2018 [26]; Pearson 2003 [138]; Philpin 2014 [15]; Remsburg 2004 [73]; Schell and Kayser-Jones 1999 [75]; Simmons 2007 [78]; Simmons and Levy-Storms 2006 [77]; Simon 2015 [79]; Sydner and Fjellström 2005 [123]; Ullrich 2011 [139]; Wang 2020 [3]; Watkins 2018 [114]; Way 2011 [92]; West 2003 [93]; Wu and Barker 2008 [80]; Wu 2015 [95]; De Wit 2020 [124]; Keller 2021 [96]; Trinca 2021 [98]; Park 2021; Morrison-Koechl 2021 [99]
Retrospective Cohort Study	28	Abbey 2015 [5]; Abbey, Wright and Capra 2015 [140]; Bamford 2012 [108]; Bennett 2015 [141]; Bertrand 2011 [53]; Buelow and Fee 2000 [55]; Bennett 2014 [22]; Caspar 2020 [83]; Chaudhury 2017b [82]; Chaudhury 2017a [81]; Carrier 2009 [1]; Evans and Crogan 2005 [12]; Gibbs-Ward and Keller 2005 [84]; Gilbart 2005 [85]; Henkusens 2014 [86]; Hung 2016 [88]; Lowndes 2018 [2]; Matwiejczyk 2018 [142]; Miles 2019 [126]; Miles 2020 [127]; Perivolaris 2006 [90]; Shune and Linville 2019 [30]; Sikorska-Simmons 2007 [76]; Steele 1997 [91]; Watkins 2017a [6]; Watkins 2019 [115]; Wu 2018 [94]; Caspar 2021 [97]
Literature Review	15	Agarwal 2016 [143]; Aselage 2011 [7]; Bellomy 2014 [51]; Chaudhury 2013 [101]; Castellanos 2004 [56]; Davis 2009 [144]; Roder-Allen 2003 [145]; Dimant 2001 [60]; Dorner 2010 [61]; Gibson and Barsade 2003 [66]; Hotaling 1990 [67]; Keller 2017 [19, 29]; Phillips and Van Ort 1995 [72]; Reimer and Keller 2009 [23]; Simmons 2007 [78]
Commentary	11	Alibrio 1991 [48]; Belardi 2014 [146]; Bowers 2014 [54]; Crogan 2001 [58]; Curtis 2008 [147]; Escott-Stump 2000 [62]; Mikula and Vanaman 2008 [71]; Minniear 1993; Stone 2014 [112]; Ullman 2009 [113]; Vivanti 2018
Systematic Review	5	Anderson 2016 [100]; Fetherstonhaugh 2019 [102]; Hines 2010 [20]; Watkins 2017b [17]; Sewell and Hopf 2020 [148]
Case Study	5	Crack and Crack 2007 [149]; Jones 2019 [110]; Keller 2015 [14]; McDonnell 2010 [70]; Roberts 2011 [74]
Ecological Study	3	Annear 2016 [130]; Aziz and Campbell-Taylor 1999 [50]; Barnes 2013 [13]
Action Research	3	Baur and Abma 2012 [116]; Byles 2009 [150]; Ullrich 2009
Evaluation	1	Benedict 1975 [52]
Government Inquiry	1	Wilson 2010 [151]
Integrative Review	1	Wang 2018 [106]
Project Report	1	Williams 2012 [107]
Book Chapter	1	Fjellström 2017 [117]
Ethical Appraisal	1	Gastmans 1998 [120]
Scoping Review	1	Vucea 2014 [105]
Case-control Study	1	Andreoli 2007 [49]
Expert Review	1	Ballesteros-Pomar 2020 [21]
Observational longitudinal study	1	Anderson and Annaliese 2021
Qualitative interviews	2	Maluf 2020 [24]; Pelletier 2005 [152]
**Total**	**137**

**Table 3:** Study settings demonstrating RACF terminology

Setting	Number of studies	Citations
Nursing Home	39	Alibrio 1991 [48]; Andreoli 2007 [49]; Aselage 2011 [7]; Bellomy 2014 [51]; Bertrand 2011 [53]; Carrier 2009 [1]; Castellanos 2004 [56]; Chang and Roberts 2008 [129]; Crogan and Evans 2001 [59]; Crogan 2001 [58]; Cohen-Mansfield 1995 [57]; Dimant 2001 [60]; Evans and Crogan 2005 [12]; Evans 2005 [63]; Evans 2003 [65]; Gastmans 1998 [120]; Grøndahl and Aagaard 2016 [25]; Harnett 2010 [118]; Harnett and Jönson 2017 [119]; Kayser-Jones and Schell 1997b [68]; Kayser-Jones 1982 [28]; McDonnell 2010 [70]; Mikula and Vanaman 2008 [71]; Milte 2017 [137]; Minniear 1993; Murphy 2017 [111]; Palacios-Cena 2013 [122]; Palese 2018 [26]; Pearson 2003 [138]; Reimer and Keller 2009 [23]; Simmons 2007 [78]; Simmons and Levy-Storms 2006 [77]; Sydner and Fjellström 2005 [123]; Wu and Barker 2008 [80]; De Wit 2020 [124]; Maluf 2020 [24]; Ballesteros-Pomar 2020 [21]; Park 2021
Nursing Centre	1	Bundgaard 2005 [4]
Skilled Nursing Facility (SNF)	4	
Care Home	10	Barnes 2013 [13]; Holmes 2019 [109]; Jones 2019 [110]; Lea 2017 [134]; Stone 2014 [112]; Ullman 2009 [113]; Ullrich 2014 [153]; Watkins 2018 [114]; Watkins 2017b [17]; Watkins 2019 [115]
Care Facility	3	Shune and Linville 2019 [30]; Anderson and Annaliese 2021; Sewell and Hopf 2020 [148]
Aged Care	7	Annear 2016 [130]; Belardi 2014 [146]; Matwiejczyk 2018 [142]; Milte 2018b [136]; Ullrich 2011 [139]; Vivanti 2018; Wilson 2010 [151]
Aged Home	1	Steele 1997 [91]
Residential Aged Care (RAC)	21	Abbey 2015 [5]; Abbey, Wright and Capra 2015 [140]; Agarwal 2016 [143]; Bennett 2014 [22]; Chisholm 2011 [125]; Chou 2002 [133]; Crack and Crack 2007 [149]; Bailey 2017 [18]; Bennett 2015 [141]; Bernoth 2014 [132]; Byles 2009 [150]; Fetherstonhaugh 2019 [102]; Hines 2010 [20]; Hogden 2017 [27]; Miles 2019 [126]; Miles 2020 [127]; Milte 2018a; Nell 2016; Wang 2020 [3]; Wang 2018 [106]; Williams 2012 [107]
Residential Care	10	Bamford 2012 [108]; Beattie 2014 [131]; Curtis 2008 [147]; Davis 2009 [144]; Roder-Allen 2003 [145]; Josefsson 2017 [121]; Philpin 2014 [15]; Watkins 2017a [6]; Maluf 2020 [24]; Caspar 2021 [97]
Residential Home	1	Baur and Abma 2012 [116]
Residential Facilities	1	Anderson 2016 [100]
Long-term Care	32	Aziz and Campbell-Taylor 1999 [50]; Caspar 2020 [83]; Chaudhury 2013 [101]; Chaudhury 2017b [82]; Chaudhury 2017a [81]; Gibbs-Ward and Keller 2005 [84]; Gibson and Barsade 2003 [66]; Gilbart 2005 [85]; Hotaling 1990 [67]; Hung and Chaudhury 2011 [87]; Hung 2016 [88]; Kayser-Jones and Schell 1997a [10]; Keller 2015 [14]; Keller 2014 [103]; Keller 2017 [19, 29]; Lengyel 2004 [89]; Lowndes 2018 [2]; Morris 2018 [104]; Perivolaris 2006 [90]; Phillips and Van Ort 1995 [72]; Remsburg 2004 [73]; Roberts 2011 [74]; Schell and Kayser-Jones 1999 [75]; Vucea 2014 [105]; Way, 2011 [92]; West 2003 [93]; Wu 2018 [94]; Wu 2015 [95]; Keller 2021 [96]; Trinca 2021 [98]; Morrison-Koechl 2021 [99]
Assisted Living	4	Buelow and Fee 2000 [55]; Mahadevan 2014 [69]; Sikorska-Simmons 2007 [76]; Simon 2015 [79]
Healthcare Organisations	1	Escott-Stump 2000 [62]
Health Care Communities	1	Dorner 2010 [61]
Formal Institutions	1	Fjellström 2017 [117]
**Total**	**137**^**a**^	

^a^The total is more than the studies included, as Maluf (2020) – 1 nursing home, 2 residential care

The conventions of Braun and Clark [154] were used to guide coding, categorising and theming. Familiarisation of the data began during full-text reviews. Codes were generated across the entirety of the 137 articles, guided by the research question and the three domains of signification, legitimation and domination, as demonstrated in Table 4. Quantitative measures relating to participant numbers, and quantitative reporting of research findings were not reflected in the codes. Multiple codes were generated for each article, and these were initially sorted according to the three domains and then further categorised into smaller sub-themes [154]. The research team refined themes and reviewed the data to establish rigour [155]. Cross-checking and fine-tuning of themes ensured that they were relevant and accurately coded under each domain (Table 4). Codes derived from articles included in the secondary search were iteratively included in the deductive analysis using constant comparison to ensure the data was grounded in the themes. No new concepts were identified from the secondary analysis. Policy documents were analysed for references to mealtime practice, processes or outcomes, and these were used to interpret the themes.

**Table 4:**
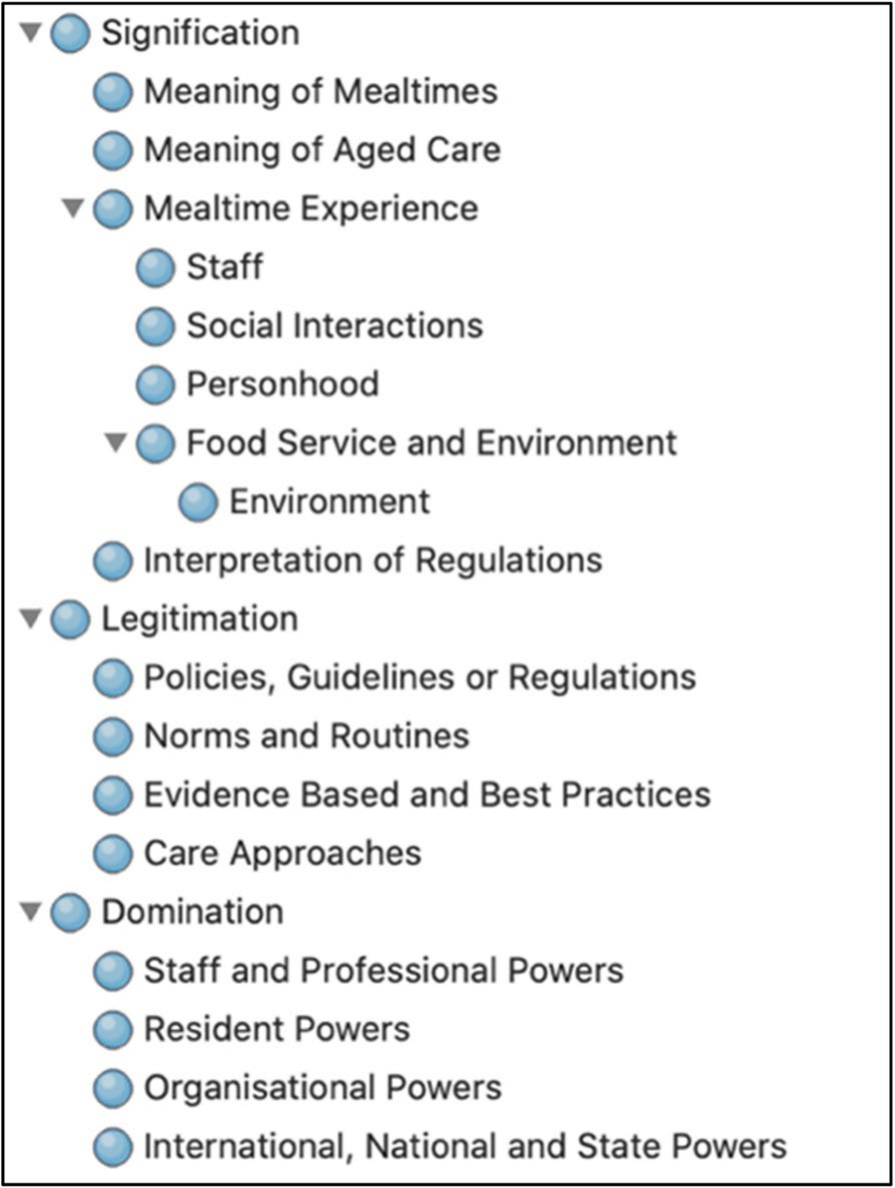
Formation of categories in each domain

## Results

### Quantitative data

As demonstrated in Tables 1 and 2, the 137 included articles yielded literature prominently from Western countries, that were most frequently cross-sectional studies reflecting diverse methods and methodologies. All articles were published between 1975 and 2021. Categorisation according to decade of publication identified that 59.1% were published after 2011, 29.9% were published between 2001 and 2010, and 10.9% were published ≤2000. Only two included studies were published prior to 1990 [28, 52].

The included articles used diverse terminology due to the different language that countries use to refer to RACFs, as demonstrated in Table 3, which charts the study settings. Full-text reviews confirmed that these terminologies were equivalent. Table 5 categorises the range of mealtime interventions and the research populations in the included literature.

**Table 5:** Range of interventions and specific study characteristics

**Intervention target**	**Interventions**	**Number of studies**	**Citations**
Environment/Setting of mealtimes	Buffet-Style Dining Program; Household dining; Enabling Person-Centred Mealtime Experiences in Residential Care Homes; Dining Environment Audit Protocol; Dining Space Renovation; CHOICE + Program; Dining space allocation; CHOICE Program	10	Andreoli 2007 [49]; Bowers 2014 [54]; Caspar 2021 [97]; Chaudhury 2017b [82]; Hung 2016 [88]; De Wit 2020 [124]; Keller 2021 [96]; Maluf 2020 [24]; Wu 2018 [94]
Care staff lead interventions	Training for resident quality of life; Best practice for residents with oro-pharyngeal dysphagia; Dining Assistance Program; Implementing Best Practice Guidelines; Feasible and Sustainable Culture Change Initiative (FASCCI) Model; Enabling Person-Centred Mealtime Experiences in Residential Care Homes; Educational Program for Food Service Providers; Situation, Background, Assessment, Recommendation approach; Paid Feeding Assistants; Making the Most of Mealtimes (M3); Protected mealtimes;	11	Anderson and Blair 2021 [156]; Ballesteros-Pomar 2020 [21]; Bertrand 2011 [53]; Byles 2009 [150]; Caspar 2020 [83]; Caspar 2021 [97]; Matwiejczyk 2018 [142]; Park 2021; Remsburg 2004 [73]; Trinca 2021 [98]; Ullrich 2011 [139]; Watkins 2019 [115]
Interventions for resident engagement, autonomy, choice	Buffet-Style Dining Program; The Taste Buddies; Enabling Person-Centred Mealtime Experiences in Residential Care Homes; FoodEx-LTC; Giving Choice of Mealtimes; Empower with Choice Program; Making the Most of Mealtimes (M3)	7	Andreoli 2007 [49]; Baur and Abma 2012 [116]; Caspar 2021 [97]; Evans and Crogan 2005 [12]; McDonnell 2010 [70]; Mikula and Vanaman 2008 [71]; Morrison-Koechl 2021 [99]; Watkins 2019 [115]
Multidisciplinary mealtime interventions	Speech Pathology Services in Residential Aged-Care Facilities; Mealtime Screening Tool;	2	Sewell and Hopf 2020 [148]; Steele 1997 [91]
Menu interventions	Best practice for residents with oro-pharyngeal dysphagia; Implementation of Nutrition Guidelines	2	Ballesteros-Pomar 2020 [21]; Bamford 2012 [108]
	**Specific study characteristics**	**Number of studies**	**Citations**
	Dementia	26	Anderson 2016 [100]; Aselage 2011 [7]; Beattie 2014 [131]; Chang and Roberts 2008 [129]; Chaudhury 2017a [81]; Roder-Allen 2003 [145]; Henkusens 2014 [86]; Hines 2010 [20]; Hung and Chaudhury 2011 [87]; Kayser-Jones 1982 [28]; Milte 2017 [137]; Murphy 2017 [111]; Nell 2016 [128]; Perivolaris 2006 [90]; Roberts 2011 [74]; Stone 2014 [112]; Way 2011 [92]; Wu 2015 [95]; De Wit 2020 [124]; Maluf 2020 [24]; Keller 2021 [96]; Caspar 2021 [97]; Trinca 2021 [98]; Anderson and Annaliese 2021; Morrison-Koechl 2021 [99]; Sewell and Hopf 2020 [148]
	Cognitive Impairment	3	Carrier 2009 [1]; Kayser-Jones and Schell 1997b [68]; Morrison-Koechl 2021 [99]
	Dysphagia	2	Shune and Linville 2019 [30]; Ballesteros-Pomar 2020 [21]

### Qualitative data

Major themes derived from each domain are described in order of their prominence in the data, illustrated by quotes and article citations. Policy information was used to interpret the themes, and was most prominent in the Legitimation and Domination domains that are more reflective of organisational practices than the Signification domain that explored the meanings residents attribute to mealtimes.

### Domain 1: signification

Four themes were captured in the Signification domain that related to the residents’ understandings and interpretations of RACF mealtimes and regulations.

#### Theme 1: mealtime experience

The most prominent theme identified, mealtime experience reflected the meanings that residents ascribed to mealtimes. These meanings were unique, formed from combined factors related to each resident’s experience. Factors included staff [83, 97, 152]; social interactions [22, 30, 99], personhood [23, 120], food service [157] and environment [16, 24] that each shape their mealtime experiences.

Mealtimes contribute to the broader RACF social environment as meanings are formed through dining interactions with staff and other residents [17]. The literature often referred to mealtimes as opportunities for social interaction that are shaped by “interactive efforts to create an appropriate version of a meal situation” ([119] p839). The mealtime meanings that residents construct are therefore influenced by how social interactions are facilitated. “The social element, meaning conversing with residents, sharing stories and feeling a sense of community, defined the meal for some residents” ([79] p35), and is associated with improved nutritional outcomes [99]. However, opportunities for residents to engage in meaningful interactions with others during mealtimes are influenced by the actions of staff, who facilitate, or do not facilitate residents’ preferences and socialisation [30, 158]. Staff also perceive a 'good meal' according to their own nutritional knowledge, training in mealtime management and personal beliefs and values [23, 152]. Thus, staff understanding of mealtime purposes and processes, influence how a resident interprets and makes meaning from mealtimes.

“Staff interpreted mealtimes in different ways. In some care homes there was little staff interaction with residents observed other than delivering the meals to the tables or rooms.” ([109] p125)

Meal delivery methods and the dining environment also influence how residents interpret and understand mealtimes [14], including interventions targeting food production and meal delivery [5], modifications to the environment, mealtime ambience and food service [12, 14, 101, 142, 150], and improving staff ratios and access to education [53, 68, 78, 112]. Additionally, moulded TMDs may improve mealtime experiences for residents with dysphagia as meaning is enhanced when food is recognisable and describable [159]. Interventions that combine environmental modifications with staff education improve mealtime experiences with greater impact than changing the physical dining space alone [90].

#### Theme 2: meaning of mealtimes

Residents bring life experiences to the RACF that inform their values and preferences. Residents with choice and control perceive mealtimes as more successful, as they can attend to their preferences about when, where and what to eat [157]. Opportunities and barriers for RACFs to promote independence and personalisation thus contribute to the mealtime meanings that residents construct [97, 158]. However, when their mealtime preferences do not align with RACF processes, residents may experience feelings of powerlessness and lost autonomy.

“Mealtimes are important opportunities to support residents’ personhood; a pleasurable dining experience affects residents’ perception of well-being and is inextricably linked with their quality of life.” ([101] p492)

Beyond nutrition, food is associated with meanings, traditions, memories and personhood, constructed across a lifetime of interactions and contexts, that shape residents’ expectations of mealtimes in the RACF [5, 117, 132]. For example, some residents see food as a symbol of security resulting from wartime austerity [133]. Their past experiences, social associations and food memories combine to structure mealtime expectations and meaning.

“Food provides more than just a way to meet the physical nutritional requirements of the body, but can also be associated with memory, social occasions, and emotions, and provide a source of enjoyment, socialisation, nurturing and dignity.” ([137] p52)

#### Theme 3: meaning of residential aged care

The literature briefly described how residents’ mealtime experiences connect with their broader understanding of their residential care experience [5, 110, 114]. A duality of structure exists, where a resident’s interpretation of mealtimes influences their RACF experience; and residing in RACFs influences the meaning they assign to mealtimes experiences. Traditionally, RACFs follow a bio-medical model [135, 144], but changing public expectations, evidence and the marketization of residential care have directed more RACFs to provide home-like environments [27]. However, many RACFs continue to view residents as care-dependent consumers with structures that institutionalise residents’ understanding of mealtimes, including mealtime schedules, menus and seating arrangements that privilege routine, standardisation and dependence [5, 24, 117].

“When a resident moves in they find the menu already set and organised and then have to adjust to being told when to eat, what meals are served and who they will be sharing a meal with in the dining room.” ([5] p36)

Acknowledging the need to shift from traditional biomedical approaches, the final report of the Royal Commission into Aged Care Quality and Safety [45] recommends future policy that incentivises the use of home-like residential care environments, and a regulatory focus on PCC practices, including mealtime practices. The final report also references the integral relationship between residents’ perceptions of quality aged care and the quality of food, the dining experience, and the implications for those who lack choice and control [160].

#### Theme 4: interpretation of regulations

Loose interpretation and different understandings of organisations and aged care accreditors about mealtime regulations were commonly reported [27, 140]. In the Australian context, the Australian Aged Care Quality Standards that provide the regulatory standards that all Australian RACFs comply with, operate on an outcome-based rather than process-oriented approach [32, 160]. Outcomes for food and nutrition care are measured using ‘unplanned weight loss’ as a single measure. Similarly, regarding meal provision, the Standards can be variably interpreted by different stakeholders, as they state that “where meals are provided, they are varied and of suitable quantity and quality” ([32] Requirement 3f), however, specific outcome measures related to this standard are lacking.

Consequently, regulators and aged care providers are permitted to variably interpret the Standards [5]. Whilst these are purported to “[provide] a mechanism by which stakeholders achieve minimum standards of quality” ([27] p140), individualised interpretations form signification structures for assessors, RACF staff and other stakeholders that influence how RACFs are rated, and how particular resident activities or care processes, such as mealtimes, are ranked for accreditation purposes. Similarly, how RACFs understand the intent of the Standards translates to the structures that guide how facilities manage and enact mealtimes, which ultimately impacts residents’ experience and their own interpretation of mealtimes [27].

### Domain 2: legitimation

The domain of legitimation captured four themes that identified the rules, processes and routines that produce structures to guide a resident’s mealtime experience.

#### Theme 1: care approaches

Care approaches in RACFs set expectations and procedures that form legitimation structures that guide the resident’s mealtime experience. PCC approaches [27, 83, 109, 110], or a social model of care [86] guide mealtime processes that “[provide] choices and preferences, supporting independence, showing respect and promoting social interaction” ([23] p327).

“In recent years, the model for long-term care settings has gone through a major paradigm shift from the traditional institutional, medical environment to more interactive communities that focus on quality of life, individual choice, and a more person-centered, home-like culture.” ([61] p1556)

Where RACFs operate under a biomedical model of care that lacks incorporation of PCC, staff may adopt a care approach that is more task-oriented than resident-focused, which impacts the mealtime experience [115]. Ultimately, the approach adopted by RACFs form structures that guide how mealtime care is enacted.

#### Theme 2: norms and routines

Mealtimes in RACFs legitimise structures related to time, place, social interactions and normality each day [4, 15]. Mealtimes provide staff with an action repertoire that also form social rituals [119]. For staff and residents, the daily routine of mealtimes often follows “an institutional script with established roles and a sequential order of action” ([119] p839), involving set timings, predetermined menus and designated resident seating [24, 86]. However, these routines provide a sense of normality and structure to the day that also benefits resident health [4, 79]. For example, saying grace is associated with initiating mealtimes [159]. Regular set menus are reported to be “imprinted into the olfactory memory” ([3] p630) of residents and their meal choices reflect comfort in familiarity and routine.

Similarly, residents’ rules and routines enacted whilst sharing food, space, company and interactions contribute to RACF mealtime structures [122]. For example, when residents deviate from the ‘code of conduct’ that directs the rules of their table, they may face admonishment from others [137]. Conversely, conventions and manners are part of proxemic behaviour that facilitate residents with dementia to participate in mealtimes [147]. Through mealtime habits and routines, residents can make sense of the broader experience of living in RACFs [24, 74]. These mealtime norms and routines provide structure for the daily activities for residents [15].

#### Theme 3: best practice

In RACFs best practice is grounded in the evidence for PCC, which informs practice guidelines and norms that form legitimation structures [18]. The literature identified some evidence supporting the assessment of mealtime needs, interventions and strategies as a mechanism to provide individualised care, optimise nutritional outcomes, and facilitate residents to enact choice and control. For example, the FoodEx-LTC assessment tool successfully identified and incorporated resident perspectives in mealtime service delivery [12]. Assessments including the ‘Dining Environment Assessment Protocol’ [161] for evaluating the physical environment and ‘Making the Most of Mealtimes’ framework [29] assist to develop and evaluate best practice mealtime interventions. Best practice assessment also requires multidisciplinary team input to develop appropriate care plans that generate new mealtime rules [141]. Additionally, open and regular communication with residents provide staff direct feedback and gauge resident expectations and experiences of meals [3].

Best practice menu guidelines for resident nutrition have been developed in Australia to direct minimum expectations for menu design, resident nutrition and intake. However, these are not mandated, and do not provide guidance about improving mealtime experiences [5, 107]. Furthermore, whilst studies have recommended policy that protects mealtimes and deters non-mealtime related tasks during meals [113, 159], these are not policy measures. A submission to ‘The Productivity Commission Public Inquiry into the Care of Older Australians’ recommended best practice guidelines to inform organisational processes and funding to improve RACF mealtimes [151, 162], and similar recommendations are provided in the Final report of the Royal Commission into Aged Care Quality and Safety [45]. Best practice recommendations form benchmarks that should set legitimation structures that underpin RACF mealtime practices, routines and actions.

#### Theme 4: policies and regulations

Policies and regulations form legitimation structures that set norms for mealtime processes and routines for staff and residents but differ between and within countries. This review most prominently derived policy guiding mealtimes from the Aged Care Quality Standards, which contain regulatory standards related to nutrition and hydration, choice and decision making, and catering, cleaning and laundry services [32, 163], but do not directly reference or measure quality mealtime practices [106].

Global regulations that direct minimum staff qualifications and care hours are lacking [5], and there are not standardised protocols or guidelines for feeding assistance, despite the relationship between eating dependency, malnutrition and complications of dysphagia [137]. Local organisational policies that govern the budget for food, staffing and time allocated for eating, vary between organisations [2, 3]. These rules and regulations direct local management and organisation of mealtimes, forming legitimation structures that translate to the practices that staff and residents enact during mealtime routines.

### Domain 3: domination

The four themes identified in the Domination domain related to power and resources individuals use to accomplish mealtime actions. These themes reflected resident, staff, organisational and government power over mealtimes.

#### Theme 1: resident power

RACFs contain domination structures that often limit the power of residents to enact control over mealtimes, and position power with the staff and institution. For example, residents lose some independence and control on entry to an RACF, including reduced access to familiar foods [3, 5]. Residents value opportunities to exercise agency and have autonomy over preferred foods, location and timing of meals, and tablemates [18], and to participate in preparing food [25].

“When asked to rate the importance of control and choice over certain areas of their everyday life in a home, residents prioritised having choice over their foods as the most important.” ([140] p7581)

However, paternalistic mealtime care approaches create domination structures that result in fewer opportunities for residents to make routine or participatory decisions [75, 86], and residents are almost entirely dependent on the facility for nourishment [67, 157]. Many residents are also aware of government policies and RACF processes that direct the extent of their choice and control at mealtimes [12], and feel resigned to having limited control [17], or are less inclined to raise concerns for fear of retribution [23, 138]. This pertains particularly to dependent residents, such as those with cognitive impairment or dysphagia, where domination structures related to care further compromise control and dignity over mealtime situations, routines and practices [21, 25, 137].

#### Theme 2: staff power

Whilst RACF staff, including nurses and nursing assistants provide the majority of mealtime interventions, dietitians, speech-language pathologists, occupational therapists and general practitioners are also directly involved in mealtime management and their interventions influence the extent that residents can exert power [141]. Staff responsibilities that include offering support to residents, fostering independence, facilitating social interactions and creating opportunities for residents to exercise autonomy can positively influence mealtime experiences [13, 109]. Whilst RACF staff have reported having little control in how RACFs operate [66], when they are empowered and “invested, aware, and knowledgeable, residents… have more individualised and ultimately, successful experiences” ([30] p149). To facilitate greater resident power requires organisations to allocate resources that enable staff to access quality education and training, and sanction practices that enable residents to enact autonomy during mealtimes [11].

#### Theme 3: organisational powers

How RACFs allocate and manage resources for mealtimes affects resident agency, mealtime culture and experience [6]. This includes fiscal and staffing constraints, such as food budget, staff workload and education [2, 131, 142]. Resource allocation strategies that relate to poor-quality mealtime experiences for residents include staff attending to non-meal tasks during mealtimes, foodservice time limitations, and cost containment schemes such as menu cycling [2, 106]. Local organisational policy further impacts on mealtime experience as this directs how the dining environment is physically managed [4]. RACFs report difficulty in balancing residents’ individual needs with organisational constraints and often prioritise the organisation’s needs [18]. Consequently, these organisational structures can impact how residents can access positive mealtimes.

Organisational support structures may enable staff to build knowledge and learn together to implement best practice and new ideas, but hierarchical staffing structures also pose barriers to staff who have ideas for improvement [134]. Organisational processes inform whether PCC is prioritised [11], and how staff enact teamwork to manage mealtimes [150], but a lack of clarity about RACF responsibilities to enact best practice has increased recommendations for multidisciplinary mealtime management to be explicitly regulated [22, 67, 109]. Thus, RACF organisational and resource allocation strategies generate domination structures related to the rules, procedures and routines that are enacted during mealtimes, and impact residents’ capacity for power and control.

#### Theme 4: government and regulatory powers

Cultural, political and economic contexts influence professional knowledge and theories on ageing which affect how RACFs are organised [4]. Governments serve as regulatory bodies to organise aged care provision by mandating regulatory policies that are tied to government funding for providers (e.g. [32, 164]). As such, through funding linked with compliance requirements, governments generate domination structures that direct the implementation of care and daily activity routines according to their priorities for funding and outcomes. However, Australian aged care policy contains no standards related to foodservice provision, mealtime quality, care or practice. Compliance measures are lacking to generate domination structures for minimum quality mealtime practices that would directly impact residents’ mealtime experiences [45].

## Discussion

This scoping review intended to identify the structures that surround and inform the mealtime experiences of RACF residents, using the Australian aged care policy context to illustrate this. The Structuration theory [34] domains of signification, legitimation and domination were used as a deductive framework, and the themes identified related to how structures facilitate and also bound the residents’ mealtime experience. Data analysis captured meanings of mealtimes, norms and traditions that inform mealtime routines and practices, regulatory and governance structures, and levels of control that residents, staff, organisations and governments have over RACF mealtimes.

This study verified that RACFs lack specific policy and regulatory structures to direct mealtime practice. The initial database searches yielded few results addressing mealtime policies and regulations, despite finding that the amount of research addressing RACF mealtime practice has substantially increased in the past decade. Existing governance and regulatory structures are more prominently directed to supporting people to select facilities, and setting general expectations of services [32, 33, 163, 165]. In Australian policy, no regulatory structure exists to direct how facilities enact mealtimes or food service despite the centrality of eating in residential life [7, 45]. Food service practices related to menu planning, food preparation, hygiene and delivery standards, staffing requirements and time allocated for mealtimes are often locally determined and vary across institutions [2, 3, 5, 113]. Domination structures set by organisations, including staff workload, time and resource pressures often legitimise task-oriented and mechanistic mealtime structures [104, 109]. This is exacerbated when staff must reconcile resident capacity to participate in mealtimes, and encourage their autonomy and independence, while maintaining work and resource efficiencies, and managing accreditation and regulatory requirements [109, 117, 149, 151]. Consequently, staff adaptive strategies often focus on adhering to care and compliance structures, i.e. what governance and organisations allow or not, which can impede PCC [117].

These structures create cognitive boundaries that limit how residents understand and experience meals, direct how mealtime practice norms and routines develop and become recursive, and inform the roles of residents and staff in enacting mealtimes according to organisational requirements [34]. Thus, structures that direct mealtime rules, routines and practices can also reduce the power of residents to enact control and autonomy [23, 156]. Furthermore, mealtime rules and practices lack transparency for residents, who are rarely consulted about foodservice processes [18, 93]. Thus, residents’ expectations of choice and control at mealtimes are not coherent with institutional processes that regulate mealtime practices and risk [3]. Synthesis from this scoping review suggests that residents, staff and policy makers must collaborate to foster a common understanding of good mealtimes to facilitate shared signification structures that improve RACF mealtime experiences.

The absence of minimum quality regulation beyond requiring “varied meals of suitable quality and quantity” ([32], Standard 4 (3f)), creates mealtime compliance issues that are particularly impactful for vulnerable populations, such as those with dysphagia or cognitive impairment, who are also more likely to depend on staff to facilitate nutrition and care outcomes [108, 127]. International guidelines exist to legitimise and standardise nutritional and swallowing practices for elderly populations, but these are inconsistently applied in RACFs. For example the *International Dysphagia Diet Standardisation Initiative* provide common and agreed terminology and standardisation for TMDs to reduce opportunities for error in meal preparation [166]. Similarly, expertise brought by professional staff supports residents and improves mealtimes. For example, dietitians who are involved in foodservice to residents and staff facilitate new mealtime signification structures by fostering understandings of nutrition that improve mealtime practice [20, 141]. However, access to professional expertise is not enshrined in policy, and compliance measures related to these services do not relate to quality outcomes [31]. Bennett and colleagues [22] contend that mealtime management must be multidisciplinary and consistent with PCC to align service provision with best practice in aged care. For this change to occur, adjustments to signification, legitimation and domination structures that direct RACF mealtimes are necessary, and must be informed by engagement and collaboration from all mealtime stakeholders.

### Implementing person-centred mealtime practice

Australian aged care policy remains silent about the separate concepts of nutrition and PCC that constitute best practice mealtimes, and has not brought these together. Conversely, whilst regulations are necessary to assure minimum quality of care, they may also impede choice and control if they hinder residents’ capacity for individualised care [57, 85]. For example, residents may not be permitted to consume foods of choice when RACFs generate menus with specified foods to meet regulatory nutritional monitoring targets [6]. It is important then that the intended and unintended consequences of policy directives to legitimise improved mealtime practices are carefully scrutinised.

Quality is currently measured through technical outcomes, and in Australian policy, ‘unexpected weight loss’ remains a proxy measure used to infer all outcomes that relate to food, mealtimes and nutrition [31]. Whilst increasing acknowledgement of consumer concerns about their rights to choice, control, and quality aged care has underpinned recent investigations into Australian aged care service provision [45], best practice in mealtime care has not yet been transformed into policy, including in recent updates of the Standards [32]. Without policy based drivers, organisational structures that legitimise current mealtime practices that do not prioritise PCC are likely to continue, as these have reproduced recursive mealtime structures despite the substantial evidence that identifies mealtime person-centred practice as integral to positive resident outcomes (e.g. [30, 83, 86, 115]).

This study identified inconsistent application of terminology and models of care in RACFs. These generate signification structures that situate different understandings and interpretations of how care is enacted that translate to legitimation and domination structures for mealtime practice. For example, the use of ‘homes’ instead of ‘facility’ or ‘institution’ connotes a social, rather than medical interpretation of care [57]. Whilst the ‘medical model’ with underlying values of individualism and reductionism legitimises structures that position care recipients as ‘objects’ who receive decisions made by professionals on their behalf, PCC models identify care recipients as ‘subjects’ with capabilities to make their own decisions [66, 94, 123]. However, Sydner and Fjellström [123] argued that viewing older people through a dichotomous lens as either “subjects” or “objects” is problematic, as individuals should be able to enact choice related to services, with appropriate staff support. In the context of mealtime practice structures, this dualistic perspective infers that residents’ capacity for agency to enact choices about menu, seating, social and environmental preferences, can be supported by policy, organisational and staff structures that respond to their needs and capabilities.

Legitimation structures that guide routinized mealtime activities and practices help to explain that the challenges confronting RACFs to incorporate PCC are greater than the policy and funding adjustments needed to set quality expectations. In RACFs, a paradoxical situation exists where routine culture is used and reproduced by both staff and residents in mutual compliance with norms and routines [24, 118]. For example, staff and resident compliance to a mandated meal time and location reinforces this practice as a norm which becomes recursive when unchallenged. When new approaches to care are introduced, staff and residents must adjust from existing norms and routines. This requires conscious engagement to interpret how new structures differ to previous routines and practice, and effort to understand and enact these outcomes. Whilst it is plausible to change care models and routines, in reality, the slow pace of change evidenced in the literature suggests that these practice and cultural changes require governance endorsement and effort from all stakeholders.

Gibson and Barsade [66] outlined a framework for RACFs to implement culture change that supports PCC: Organisations must firstly develop an understanding of good mealtimes and critically compare this with current practice to generate new meanings. These new signification structures underpin goals, actions and outcomes, including setting parameters and quality indicators of successful and person-centred mealtimes. Strong leadership is needed to intentionally align internal structures, systems and policies with person-centred mealtimes, and develop governance structures that empower residents and staff to reflexively monitor mealtime practices, and modify structures to afford best practice outcomes [34, 88, 115]. For example, policy adjustments to enable staffing, meal delivery and foodservice systems, or to flatten organisational structures may be required to legitimise staff to respond effectively to residents during mealtimes [115, 144, 150]. The enactment of this framework enables the structures that direct mealtimes to be modified, and facilitates person-centred practices that relate to “good mealtimes” to become routinized and normalised.

## Limitations

Structuration Theory is most often applied as a practice theory to explore the activities that unfold, are produced and then become reproduced within a particular social context [34]. Whilst the notion of a scoping review suggests a context-free analysis, this review was situated within the policy and governance frameworks of the Australian aged-care context. Thus, in respect to literature constructed and gathered from a broad global context, Structuration theory provided a framework to examine how the Australian policy context generates and reproduces RACF mealtime practices. We acknowledge that in using the Australian policy context as a lens to explore mealtime practices, we have assumed both that international evidence gathered and analysed through the scoping review framework is or could be applied in the situation of Australian RACFs; and that social practices, which are known to be contextually bound, are similar across Australian RACFs. However, this does not attend to the broad range of RACF settings, even within Australia, which vary according to local sociodemographic, geographical, cultural and funding factors [45]; and these assumptions are limitations of this study.

The use of Structuration Theory as a framework to explore how the Australian policy and governance context impacts mealtime practices in RACFs is, in itself, limiting, as the analysis is blind to concepts that are absent from the theory. For example, whilst this study explored notions of residents and staff as agents who operate within mealtime practice structures, it did not explore the social or cultural capital that individuals bring to their agency [167], which may be an important lever of mealtime practice and outcomes that alternative theoretical framing may open up.

As this study was limited to the Australian policy and governance context, it is not known if recommendations from this study can be generalised to the international context as this review identified that structures, policy and terminology that reflect aged care practices vary globally. The literature included in this review was strongly grounded in Western countries, and the structures that guide meaning-making, and are legitimised and sanctioned in these contexts may not translate to broader global contexts. Indeed, the different ethnic, cultural, sociodemographic and political contexts of global aged care may necessitate variations in mealtime practice and how this translates to the experiences and meanings that residents ascribe to mealtimes. This prompts the need for future research exploring global RACF mealtime contexts and how “good mealtimes” translate for ethnically, culturally and geographically diverse residents. However, this study provides a useful case study about how structures generated by policy and governance procedures in an Australian context directly and indirectly affect the mealtime experience of RACF residents.

## Conclusion

This scoping review used Structuration Theory [34] as an interpretative lens to investigate how policies and regulatory guidelines translate into practice during mealtimes in RACFs. Data analysis identified structures that direct mealtime experiences for residents, examined the relationship between these structures and the resulting actions of residents and staff, and how these actions are informed by government regulations and organisational policy and procedures.

Current policy lacks specificity and is limited in informing structures and practices of RACF mealtimes. These inadequate regulatory and funding structures do not provide sufficient guidance to facilitate quality, person-centred mealtime experiences for RACF residents, despite this approach being best practice. Furthermore, residents, staff and policy-makers possess different signification, legitimation and domination structures that guide the meaning and practice of meals, and this lack of shared understanding is likely to negatively impact the residents’ mealtime experience. This is perpetuated by domination structures that guide mealtime resource allocation and staff structures, and these direct the capacity and accountability for person-centred mealtime practices. Organisational and cultural changes are required to align service provision with PCC, facilitate shared understanding, and translate these into positive practice changes that improve residents’ mealtime experiences, and ultimately their QoL.

## Supplementary Information

**Additional file 1.** Population, Concept, Context Terms Generated.

**Additional file 2.** Specific Search Terms.

**Additional file 3.** Number of Results from Database and Engine Search.

## Data Availability

All data generated or analysed during this study are included in this published article (and supplementary information files).

## References

[1] Carrier N, West GE, Ouellet D (2009). Dining experience, foodservices and staffing are associated with quality of life in elderly nursing home residents. J Nutr Health Aging.

[2] Lowndes R, Daly T, Armstrong P (2018). “Leisurely dining”: exploring how work organization, informal care, and dining spaces shape residents’ experiences of eating in long-term residential care. Qual Health Res.

[3] Wang D (2020). Perspectives of residents and staff regarding food choice in residential aged care: a qualitative study. J Clin Nurs.

[4] Bundgaard KM. The meaning of everyday meals in living units for older people. J Occup Sci. 2005;12(2):91-101. 10.1080/14427591.2005.9686552.

[5] Abbey KL. Australian residential aged care foodservices menu design, quality and standards-a time for action. Queensland: University of Queensland; 2015.

[6] Watkins R (2017). Attitudes, perceptions and experiences of mealtimes among residents and staff in care homes for older adults: a systematic review of the qualitative literature. Geriatr Nurs.

[7] Aselage MB, Amella EJ, Watson R (2011). State of the science: alleviating mealtime difficulties in nursing home residents with dementia. Nurs Outlook.

[8] Speroff BA (2005). The dining experience in nursing homes. N C Med J.

[9] Hodgkin S (2017). Workforce crisis in residential aged care: insights from rural, older workers. Aust J Public Adm.

[10] Kayser-Jones J, Schell ES (1997). Staffing and the mealtime experience of nursing home residents on a special care unit. Am J Alzheimers Dis.

[11] Keller HH (2021). Relationship-centered mealtime training program demonstrates efficacy to improve the dining environment in long-term care. J Am Med Dir Assoc.

[12] Evans BC, Crogan NL (2005). Using the FoodEx-LTC to assess institutional food service practices through nursing home residents’ perspectives on nutrition care. J Gerontol A Biol Sci Med Sci.

[13] Barnes S (2013). Exploring the mealtime experience in residential care settings for older people: an observational study. Health Soc Care Community.

[14] Keller H, Beck AM, Namasivayam A (2015). Improving food and fluid intake for older adults living in long-term care: a research agenda. J Am Med Dir Assoc.

[15] Philpin S, Merrell JOY, Warring J, Hobby D, Gregory VIC. Memories, identity and homeliness: the social construction of mealtimes in residential care homes in South Wales. Ageing amp; Society. 2014;34(5):753–89. 10.1017/S0144686X12001274.

[16] Kenkmann A, Hooper L (2012). The restaurant within the home: experiences of a restaurant-style dining provision in residential homes for older people. Qual Ageing Older Adults.

[17] Watkins R (2017). Exploring residents’ experiences of mealtimes in care homes: a qualitative interview study. BMC Geriatr.

[18] Bailey A, Bailey S, Bernoth M (2017). ‘I’d rather die happy’: residents’ experiences with food regulations, risk and food choice in residential aged care. A qualitative study. Contemp Nurse.

[19] Keller HH (2017). Prevalence and determinants of poor food intake of residents living in long-term care. J Am Med Dir Assoc.

[20] Hines S (2010). Thickened fluids for people with dementia in residential aged care facilities. JBI Evid Implement.

[21] Ballesteros-Pomar MD (2020). Texture-modified diet for improving the management of oropharyngeal dysphagia in nursing home residents: an expert review. J Nutr Health Aging.

[22] Bennett MK (2014). Perspectives on mealtime management in residential aged care: insights from a cross-disciplinary investigation. J Nutr Gerontol Geriatr.

[23] Reimer HD, Keller HH (2009). Mealtimes in nursing homes: striving for person-centered care. J Nutr Elder.

[24] Maluf A (2020). Structure and agency attributes of residents’ use of dining space during mealtimes in care homes for older people. Health Soc Care Community.

[25] Grøndahl VA, Aagaard H (2016). Older people’s involvement in activities related to meals in nursing homes. Int J Older People Nursing.

[26] Palese A (2018). Interventions maintaining eating Independence in nursing home residents: a multicentre qualitative study. BMC Geriatr.

[27] Hogden A (2017). How does accreditation influence staff perceptions of quality in residential aged care?. Qual Ageing Older Adults.

[28] Kayser-Jones JS (1982). Institutional structures: catalysts of or barriers to quality care for the institutionalized aged in Scotland and the U.S. Soc Sci Med.

[29] Keller HH (2017). Making the most of mealtimes (M3): protocol of a multi-centre cross-sectional study of food intake and its determinants in older adults living in long term care homes. BMC Geriatr.

[30] Shune SE, Linville D (2019). Understanding the dining experience of individuals with dysphagia living in care facilities: a grounded theory analysis. Int J Nurs Stud.

[31] Aged Care Quality and Safety Commission (2019). National aged care mandatory quality indicator program, D.o. Health, editor.

[32] Aged Care Quality and Safety Commission. Quality standards, Department of Health, editor. Canberra: Commonwealth of Australia; 2020.

[33] Aged Care Quality and Safety Commission. Commission Act and Rules. Canberra: Department of Health; 2020.

[34] Giddens A. The constitution of society: outline of the theory of structuration. USA: University of California Press; 1984.

[35] Varpio L (2020). The distinctions between theory, theoretical framework, and conceptual framework. Acad Med.

[36] Peters MDJ, Aromataris E, Munn Z (2020). Chapter 11: scoping reviews. JBI manual for evidence synthesis.

[37] Arksey H, O’Malley L (2005). Scoping studies: towards a methodological framework. Int J Soc Res Methodol.

[38] Haddaway NR (2015). The role of Google Scholar in evidence reviews and its applicability to grey literature searching. PLoS One.

[39] The EndNote Team (2013). EndNote.

[40] Covidence (2020). Covidence systematic review software.

[41] McHugh ML (2012). Interrater reliability: the kappa statistic. Biochem Med.

[42] Waffenschmidt S (2019). Single screening versus conventional double screening for study selection in systematic reviews: a methodological systematic review. BMC Med Res Methodol.

[43] Moher D, Liberati A, Tetzlaff J, Altman DG, The PRISMA Group (2009). Preferred reporting items for systematic reviews and meta-analyses: the PRISMA statement. PLoS Med (Open Access).

[44] Lewis S (2015). Qualitative inquiry and research design: choosing among five approaches. Health Promot Pract.

[45] Commonwealth of Australia (2021). Royal Commission into aged care quality and safety. Final report: care, dignity and respect.

[46] QSR International Pty Ltd (2018). NVivo (version 12).

[47] Adams K, Anderson JB, Archuleta M, Smith Kudin J. Defining skilled nursing facility residents’ dining style preferences. J Nutr Gerontol Geriatr. 2013;32(3):213–32. 10.1080/21551197.2013.810560. 10.1080/21551197.2013.810560 23924255

[48] Alibrio T. Food: An important focus for nursing home residents. Nursing Homes, 40(2), 9-10. evidence. Int Psychogeriatr. 1991;28(8):1263–81.

[49] Andreoli NA, Breuer L, Marbury D, Williams S, Rosenblut MN. Serving Culture Change at Mealtimes: Residents fare better medically and socially in a person-centered dining environment. Nursing Homes-Washington then Cleveland; 2007;56(9):48.

[50] Aziz SJ, Campbell-Taylor I. Neglect and abuse associated with undernutrition in long-term care in North America: Causes and solutions. J Elder Abuse Negl. 1999;10(1-2):91–117. 10.1300/J084v10n01_07.

[51] Bellomy, K. Successful mealtimes for persons with dementia in nursing homes. 2014. 10.17615/8vtt-fz72.

[52] Benedict S (1975). Medical care evaluation studies: for utilization review in skilled nursing facilities.

[53] Bertrand RM (2011). The nursing home dining assistant program: a demonstration project. J Gerontol Nurs.

[54] Bowers LA. Household dining yields lower costs, higher satisfaction. Long-Term Living. 2014;63(4).

[55] Buelow JR, Fee FA. Perceptions of care and satisfaction in assisted living facilities. Health marketing quarterly. 2000;17(3):13-24. 10.1300/J026v17n03_02 11010217

[56] Castellanos VH. Food and nutrition in nursing homes. Generations. 2004;28(3):65-71.

[57] Cohen-Mansfield J (1995). Autonomy for nursing home residents: the role of regulations. Behav Sci Law.

[58] Crogan NL, Shultz JA, Adams CE, Massey LK. Barriers to nutrition care for nursing home residents. J Geriatr Nurs. 2001;27(12):25–31. 10.3928/0098-9134-20011201-09 11820531

[59] Crogan NL, Evans B. Guidelines for improving resident dining experiences in long-term care facilities. J Nurses Prof Dev. 2001;17(5):256–59. 10.1097/00124645-200109000-00009 12759995

[60] Dimant J. Delivery of nutrition and hydration care in nursing homes: Assessment and interventions to prevent and treat dehydration, malnutrition, and weight loss. J Am Med Dir Assoc. 2001;2(4):175-82. 10.1016/S1525-8610(04)70196-6. 12812577

[61] Dorner B (2010). Practice paper of the American dietetic association: individualized nutrition approaches for older adults in health care communities. J Am Diet Assoc.

[62] Escott-Stump S, Krauss B, Pavlinac J, Robinson G. Joint Commission on Accreditation of Healthcare Organizations: Friend, Not Foe. J Acad Nutr Diet. 2000;100(7):839. 10.1016/S0002-8223(00)00243-1 10916527

[63] Evans BC, Crogan NL, Shultz JA. The meaning of mealtimes: connection to the social world of the nursing home. J Geriatr Nurs. 2005;31(2):11–17. 10.3928/0098-9134-20050201-05. 10.3928/0098-9134-20050201-05 15756981

[64] Evans BC, Crogan NL, Shultz JA. Resident coping strategies in the nursing home: An indicator of the need for dietary services change. Appl Nurs Res. 2004;17(2):109-15. 10.1016/j.apnr.2004.02.003. 10.1016/j.apnr.2004.02.003 15154123

[65] Evans BC, Crogan NL, Shultz JA. Quality dining in the nursing home: the residents' perspectives. J Nutr Elderly. 2003;22(3):1–17. 10.1300/J052v22n03_01.

[66] Gibson DE, Barsade SG (2003). Managing organizational culture change. J Soc Work Long Term Care.

[67] Hotaling DL (1990). Adapting the mealtime environment: setting the stage for eating. Dysphagia.

[68] Kayser-Jones J, Schell E (1997). The mealtime experience of a cognitively impaired elder: ineffective and effective strategies. J Gerontol Nurs.

[69] Mahadevan M, Hartwell HJ, Feldman CH, Ruzsilla JA, Raines ER. Assisted‐living elderly and the mealtime experience. J Hum Nutr Diet. 2014;27(2):152-61. 10.1111/jhn.12095. 10.1111/jhn.12095 23489649

[70] McDonnell K. Testing the theory. Activities, Adaptation amp; Aging. 2010;34(4):314-16. 10.1080/01924788.2010.534945.

[71] Mikula J, Vanaman T. Empower with choice. Long Term Living Magazine. 2008;9:30-43. https://hdl.handle.net/10365/28704.

[72] Phillips LR, Van Ort S. Issues in conducting intervention research in long-term care settings. Nursing Outlook. 1995;43(6):249-53. 10.1016/S0029-6554(95)80089-1. 10.1016/s0029-6554(95)80089-1 8668558

[73] Remsburg RE. Pros and cons of using paid feeding assistants in nursing homes. Geriatric Nursing. 2004;25(3):176-77. 10.1016/j.gerinurse.2004.04.009 15197379

[74] Roberts E (2011). Six for lunch: a dining option for residents with dementia in a special care unit. J Hous Elder.

[75] Schell ES, Kayser-Jones J (1999). The effect of role-taking ability on caregiver-resident mealtime interaction. Appl Nurs Res.

[76] Sikorska-Simmons E. The effects of organizational policies on resident perceptions of autonomy in assisted living. J Hous Elderly. 2007;20(4):61-77. 10.1300/J081v20n04_05.

[77] Simmons SF, Levy-Storms L. The Effect of Staff Care Practices on Nursing Home Residents' preferences: Implications for Individualizing Care. The Journal of nutrition, health amp; aging. 2006;10(3):216. 16622583

[78] Simmons SF (2007). A preliminary evaluation of the paid feeding assistant regulation: impact on feeding assistance care process quality in nursing homes. The Gerontologist.

[79] Simon M (2015). The mealtime experience in independent and assisted living centers: a qualitative study.

[80] Wu S, Barker JC. Hot tea and juk: the institutional meaning of food for Chinese elders in an American nursing home. J Gerontol Nurs. 2008;34(11):46-54. 10.3928/00989134-20081101-11. 10.3928/00989134-20081101-11 19024429

[81] Chaudhury H, Keller H, Pfisterer K, Hung L. Development of a physical environmental observational tool for dining environments in long-term care settings. The Gerontologist. 2017a;57(6):e95-e101. 10.1093/geront/gnw261. 10.1093/geront/gnw261 28329819

[82] Chaudhury H, Hung L, Rust T, Wu S. Do physical environmental changes make a difference? Supporting person-centered care at mealtimes in nursing homes. Dementia. 2017b;16(7):878-96. 10.1177/1471301215622839. 10.1177/1471301215622839 26764264

[83] Caspar S, et al. Staff engagement for practice change in long-term care: evaluating the feasible and sustainable culture change initiative (FASCCI) model. J Long Term Care. 2020:30–41. 10.31389/jltc.25.

[84] Gibbs-Ward AJ, Keller HH. Mealtimes as active processes in long-term care facilities. Can J Diet Pract Res. 2005;66(1):5-11. 10.3148/66.1.2005.5. 10.3148/66.1.2005.5 15780150

[85] Gilbart E. Conceptual model of clinical practice guideline implementation: case study of long-term care in Ontario. In: UMI dissertation services, ProQuest information and learning. Ann Arbor; 2005.

[86] Henkusens C (2014). Transitions to long-term care:how do families living with dementia experience mealtimes after relocating?. J Appl Gerontol.

[87] Hung L, Chaudhury H. Exploring personhood in dining experiences of residents with dementia in long-term care facilities. J Aging Stud. 2011;25(1):1–12. 10.1016/j.jaging.2010.08.007.

[88] Hung L, Chaudhury H, Rust T (2016). The effect of dining room physical environmental renovations on person-centered care practice and residents’ dining experiences in long-term care facilities. J Appl Gerontol.

[89] Lengyel CO, Smith JT, Whiting SJ, Zello GA. A questionnaire to examine food service satisfaction of elderly residents in long-term care facilities. J Nutr Elderly. 2004;24(2):5-18. 10.1300/J052v24n02_02. 10.1300/J052v24n02_02 15778154

[90] Perivolaris A (2006). An enhanced dining program for persons with dementia. Alzheimers Care Today.

[91] Steele CM, Greenwood C, Ens I, Robertson C, Seidman-Carlson R. Mealtime difficulties in a home for the aged: not just dysphagia. Dysphagia. 1997;12(1):43-50. 10.1007/pl00009517 8997832

[92] Way C. " Place" and the Mealtime Experience for those Living with Dementia: Transitions to Long-term Care (Doctoral dissertation). 2011. http://hdl.handle.net/10214/2891.

[93] West GE, Ouellet D, Ouellette S (2003). Resident and staff ratings of foodservices in long-term care: implications for autonomy and quality of life. J Appl Gerontol.

[94] Wu S (2018). Mixed methods developmental evaluation of the CHOICE program: a relationship-centred mealtime intervention for long-term care. BMC Geriatr.

[95] Wu SA. Exploring person-centered care and mealtimes for residents with dementia in specialized care units (Doctoral dissertation, Arts amp; Social Sciences: Department of Gerontology). 2015.

[96] Keller HH, et al. Relationship-Centered Mealtime Training Program Demonstrates Efficacy to Improve the Dining Environment in Long-Term Care. J Am Med Dir Assoc. 2021;22(9):1933-1938.e2. 10.1016/j.jamda.2020.11.008 33306996

[97] Caspar S (2021). Stakeholder engagement in practice change: enabling person-centred mealtime experiences in residential care homes. Can J Aging.

[98] Trinca V, et al. Making the Most of Mealtimes (M3): Association Between Relationship-Centered Care Practices, and Number of Staff and Residents at Mealtimes in Canadian Long-Term Care Homes. J Am Med Dir Assoc. 2021;22(9):1927-1932.e1. 10.1016/j.jamda.2020.11.020 33338445

[99] Morrison-Koechl J (2021). Hungry for more: low resident social engagement is indirectly associated with poor energy intake and mealtime experience in long-term care homes. Appetite.

[100] Anderson K, Bird M, MacPherson S, Blair A. How do staff influence the quality of long-term dementia care and the lives of residents? A systematic review of the evidence. Int Psychogeriatr. 2016;28(8):1263–81. 10.1017/S1041610216000570. 10.1017/S1041610216000570 27082717

[101] Chaudhury H, Hung L, Badger M (2013). The role of physical environment in supporting person-centered dining in long-term care:a review of the literature. Am J Alzheimers Dis Other Demen.

[102] Fetherstonhaugh D, Haesler E, Bauer M. Promoting mealtime function in people with dementia: A systematic review of studies undertaken in residential aged care. Int J Nurs Stud. 2019;96:99-118. 10.1016/j.ijnurstu.2019.04.005. 10.1016/j.ijnurstu.2019.04.005 31060734

[103] Keller H, Carrier N, Duizer L, Lengyel C, Slaughter S, Steele C. Making the Most of Mealtimes (M3): grounding mealtime interventions with a conceptual model. J Am Med Dir Assoc. 2014;15(3):158-61. 10.1016/j.jamda.2013.12.001. 10.1016/j.jamda.2013.12.001 PMC4316206 24513225

[104] Morris JN (2018). Hearing the voice of the resident in long-term care facilities—an internationally based approach to assessing quality of life. J Am Med Dir Assoc.

[105] Vucea V, Keller HH, Ducak K. Interventions for improving mealtime experiences in long-term care. J Nutr Gerontol Geriatr. 2014;33(4):249-324. 10.1080/21551197.2014.960339. 10.1080/21551197.2014.960339 25424508

[106] Wang D (2018). Access to food choices by older people in residential aged care: an integrative review. Collegian.

[107] Williams P (2012). Development of nutrition and menu planning standards for residential aged care facilities in Australia and New Zealand.

[108] Bamford C (2012). Implementing nutrition guidelines for older people in residential care homes: a qualitative study using normalization process theory. Implement Sci.

[109] Holmes J (2019). An exploration of the factors that affect the extensive meal experience for the older person living in residential care.

[110] Jones M, Ismail SU, Whole A. Setting approach to food in care homes. A case study evaluation of the food for life better care programme in Calderdale. Bristol: UWE Bristol; 2019.

[111] Murphy JL, Holmes J, Brooks C. Nutrition and dementia care: developing an evidence-based model for nutritional care in nursing homes. BMC geriatrics. 2017;17(1):1-14. 10.1186/s12877-017-0443-2. 10.1186/s12877-017-0443-2 PMC5309970 28196475

[112] Stone L (2014). Eating/feeding issues in dementia: improving the dining experience. End Life J.

[113] Ullman S (2009). The contribution of care home staff to nutrition and hydration. Nurs Resid Care.

[114] Watkins R (2018). Eating well: understanding and shaping the mealtime experience of older adults in residential care.

[115] Watkins R (2019). Eating well in care homes: testing the feasibility of a staff training programme aimed at improving social interaction and choice at mealtimes. Int J Older People Nursing.

[116] Baur VE, Abma TA. 'The Taste Buddies': participation and empowerment in a residential home for older people. Ageing and society. 2012;32(6):1055-78. http://journals.cambridge.org/abstract_S0144686X11000766.

[117] Fjellström C, Sydner YM. Social Significance of Older People’s Meals—Balancing Adaptive Strategies Between Ideals and Structure. In Food for the Aging Population (pp. 83-98). Woodhead Publishing. 2017. 10.1016/B978-0-08-100348-0.00004-4.

[118] Harnett T (2010). Seeking exemptions from nursing home routines: residents’ everyday influence attempts and institutional order. J Aging Stud.

[119] Harnett T, Jönsson H (2017). Shaping nursing home mealtimes. Ageing Soc.

[120] Gastmans C (1998). Meals in nursing homes. Scand J Caring Sci.

[121] Josefsson MS, Nydahl M, Persson I, Sydner YM. Quality indicators of nutritional care practice in elderly care. J Nutr Health Aging. 2017;21(9):1057-64. 10.1007/s12603-017-0970-8. 10.1007/s12603-017-0970-8 PMC5662708 29083448

[122] Palacios-Ceña D (2013). Is the mealtime experience in nursing homes understood? A qualitative study. Geriatr Gerontol Int.

[123] Sydner YM, Fjellström C (2005). Food provision and the meal situation in elderly care – outcomes in different social contexts. J Hum Nutr Diet.

[124] De Wit Y. Mealtime care for nursing home residents. Ghent University; 2020.

[125] Chisholm A, Jensen J, Field P. Eating environment in the aged‐care residential setting in New Zealand: Promoters and barriers to achieving optimum nutrition. Observations of the foodservice, menu and meals. Nutr Diet. 2011;68(2):161-66. 10.1111/j.1747-0080.2011.01510.x.

[126] Miles A, Dennison K, Oad MA, Shasha L, Royal M. Consumer Satisfaction of Texture Modified Meals Served in Residential Aged-Care Facilities. 2019. 10.31546/IJFSNR.1005.

[127] Miles A (2020). Texture-modified diets in aged care facilities: nutrition, swallow safety and mealtime experience. Aust J Ageing.

[128] Nell D, Neville S, Bellew R, O'Leary C, Beck KL. Factors affecting optimal nutrition and hydration for people living in specialised dementia care units: A qualitative study of staff caregivers' perceptions. Australas J Ageing. 2016;35(4):E1-E6. 10.1111/ajag.12307. 10.1111/ajag.12307 26969881

[129] Chang CC, Roberts BL. Cultural perspectives in feeding difficulty in Taiwanese elderly with dementia. J Nurs Scholarsh. 2008;40(3):235-40. 10.1111/j.1547-5069.2008.00231.x. 10.1111/j.1547-5069.2008.00231.x 18840206

[130] Annear MJ, Otani J, Sun J. Experiences of Japanese aged care: the pursuit of optimal health and cultural engagement. Age and Ageing. 2016;45(6):753-56. 10.1093/ageing/afw144. 10.1093/ageing/afw144 27506440

[131] Beattie E (2014). How much do residential aged care staff members know about the nutritional needs of residents?. Int J Older People Nursing.

[132] Bernoth MA, Dietsch E, Davies C (2014). Two dead frankfurts and a blob of sauce: the serendipity of receiving nutrition and hydration in Australian residential aged care. Collegian.

[133] Chou S-C, Boldy DP, Lee AH (2002). Resident satisfaction and its components in residential aged care. The Gerontologist.

[134] Lea EJ (2017). Staff awareness of food and fluid care needs for older people with dementia in residential care: a qualitative study. J Clin Nurs.

[135] Milte R, Ratcliffe J, Chen G, Miller M, Crotty M. Taste, choice and timing: Investigating resident and carer preferences for meals in aged care homes. Nurs Health Sci. 2018a;20(1):116-24. 10.1111/nhs.12394. 10.1111/nhs.12394 PMC6635740 29314590

[136] Milte R, Bradley C, Miller M, Farrer O, Crotty M. How Widely are Supportive and Flexible Food Service Systems and Mealtime Interventions Used for People in Residential Care Facilities? A Comparison of Dementia-Specific and Nonspecific Facilities. In Healthcare (Vol. 6, No. 4, p. 140). Multidisciplinary Digital Publishing Institute. 2018b. 10.3390/healthcare6040140. 10.3390/healthcare6040140 PMC6316499 30513902

[137] Milte R (2017). Struggling to maintain individuality – describing the experience of food in nursing homes for people with dementia. Arch Gerontol Geriatr.

[138] Pearson A, Fitzgerald M, Nay R (2003). Mealtimes in nursing homes: the role of nursing staff. J Gerontol Nurs.

[139] Ullrich S, McCutcheon H, Parker B. Reclaiming time for nursing practice in nutritional care: outcomes of implementing Protected Mealtimes in a residential aged care setting. J Clin Nurs. 2011;20(9‐10):1339-48. 10.1111/j.1365-2702.2010.03598.x. 10.1111/j.1365-2702.2010.03598.x 21492280

[140] Abbey KL, Wright ORL, Capra S (2015). Menu planning in residential aged care—the level of choice and quality of planning of meals available to residents. Nutrients.

[141] Bennett MK, Ward EC, Scarinci NA (2015). Mealtime management in Australian residential aged care: comparison of documented, reported and observed care. Int J Speech Lang Pathol.

[142] Matwiejczyk L (2018). Engaging food service providers to change food service practices in aged care facilities. Nutr Diet.

[143] Agarwal E, et al. Optimising nutrition in residential aged care: A narrative review. Maturitas. 2016;92:70-8. 10.1016/j.maturitas.2016.06.013 27621242

[144] Davis S (2009). Guiding design of dementia friendly environments in residential care settings: considering the living experiences. Dementia.

[145] Roder-Allen G, Willick C, De Bellis A, Mitchell P. Food for thought: residents with dementia who require assistance with eating and drinking. Geriaction. 2003;21(3):5–10.

[146] Belardi, L. A simmering issue. Australian Ageing Agenda. 2014;57.

[147] Curtis K. Nutrition: manners maketh the meal. Australia: The Intermedia Group; 2008. p. 98–9.

[148] Sewell SA, SC. Hopf, Speech-language pathology in Australian residential aged-care facilities. Journal of clinical practice in speechlanguage pathology. 2020;22(1):53-61.

[149] Crack J, Crack G (2007). Promoting quality care for older people in meal management: whose responsibility is it?. Aust Nurs Midwifery Fed.

[150] Byles J (2009). Encouraging best practice nutrition and hydration in residential aged care. Final report., D.o.H. Ageing, editor.

[151] Wilson J (2010). Productivity Commission public inquiry into the care of older Australians: submission.

[152] Pelletier CA (2005). Feeding beliefs of certified nurse assistants in the nursing home: a factor influencing practice. J Gerontol Nurs.

[153] Ullrich S, Buckley J, Crichton J, Esterman A. An Exploratory Study of the Mealtime Experience of Older People with Dysphagia (Doctoral dissertation, SERDI). Vivanti, A. (2018). Improving the quality of life of aged care residents through the joy of food. Australas J Ageing. 2014;37(4):252-53. 10.1111/ajag.12510. 10.1111/ajag.12530 30506816

[154] Braun V, Clarke V (2006). Using thematic analysis in psychology. Qual Res Psychol.

[155] Patton MQ. Qualitative research & evaluation methods. UK: SAGE Publications; 2002.

[156] Anderson K, Blair A. What have staff got to do with it? Untangling complex relationships between residential aged care staff, the quality of care they provide, and the quality of life of people with dementia. Arch Gerontol Geriatr. 2021;94:104378. 10.1016/j.archger.2021.104378 33631693

[157] Evans BC, Crogan NL, Shultz JA (2003). Quality dining in the nursing home. J Nutr Elder.

[158] Trinca V (2021). Making the most of mealtimes (M3): association between relationship-centered care practices, and number of staff and residents at mealtimes in Canadian long-term care homes. J Am Med Dir Assoc.

[159] Ullrich S (2014). An exploratory study of the mealtime experience of older people with dysphagia.

[160] Commonwealth of Australia. Royal Commission into aged care quality and safety. Canberra; 2020.

[161] Chaudhury H (2017). Development of a physical environmental observational tool for dining environments in long-term care settings. The Gerontologist.

[162] Productivity Commission. Caring for older Australians: overview, in final inquiry report. Canberra; 2011.

[163] Aged Care Quality and Safety Commission (2020). Old standards, Department of Health, editor.

[164] Australian Government. Aged care quality and safety commission act 2018. Australia; 2018.

[165] Aged Care Quality and Safety Commission. Charter of Aged Care Rights. Canberra: Department of Health; 2020.

[166] International Dysphagia Diet Standardisation Initiative (2020). What is the IDDSI framework?.

[167] Bourdieu P, Richardson J (1986). The forms of capital in handbook of theory and research for the sociology of education.

