# Supplementary material for: How are the mealtime experiences of people in residential aged care facilities informed by policy and best practice guidelines? A scoping review
Source: BMC Geriatr. 2022 Sep 9;22:737. doi: 10.1186/s12877-022-03340-9 (PMC9463738; doi:10.1186/s12877-022-03340-9)
Supplement: Supplementary file 1 — Additional file 1. Population, Concept, Context Terms Generated. [file 12877_2022_3340_MOESM1_ESM.docx]

**Appendix A: Population, Concept, Context Terms Generated**

|  | **Terms** |
| --- | --- |
| **Population** | Aged, geriatric, elderly |
| **Concept** | (a) experience, attitude, opinion, view, feeling, perspective, perception  (b) policies, evidence, guidelines, processes, structures, best practice, governance, protocols, accreditation, standards, quality (of life) |
| **Context** | (a) mealtimes, eating, feeding, swallowing, dysphagia, nutrition, food intake  (b) residential aged care facilities, care homes, high level care, long term care facilities, skilled nursing facilities |
