# Supplementary material for: How are the mealtime experiences of people in residential aged care facilities informed by policy and best practice guidelines? A scoping review
Source: BMC Geriatr. 2022 Sep 9;22:737. doi: 10.1186/s12877-022-03340-9 (PMC9463738; doi:10.1186/s12877-022-03340-9)
Supplement: Supplementary file 2 — Additional file 2. Specific Search Terms. [file 12877_2022_3340_MOESM2_ESM.docx]

**Appendix B: Specific Search Terms**

1. **Ageline**
   1. **Search A**

| # | Query | Limiters/Expanders | Results |
| --- | --- | --- | --- |
| 1 | meal* OR mealtime OR breakfast OR lunch OR dinner OR tea OR eat* OR feed* OR feeding practices OR swallowing OR dysphagia OR nutrition | Expanders - Apply equivalent subjects  Search modes - Boolean/Phrase | 9353 |
| 2 | food intake | Expanders - Apply equivalent subjects  Search modes - Boolean/Phrase | 1218 |
| 3 | S1 OR S2 | Expanders - Apply equivalent subjects  Search modes - Boolean/Phrase | 9425 |
| 4 | experience* OR attitude* OR opinion* OR view* OR feeling* OR perspective* OR perception* | Expanders - Apply equivalent subjects  Search modes - Boolean/Phrase | 58610 |
| 5 | residential aged care facilit* OR aged care OR residential care OR care home* OR high level care OR long term care facilit* OR skilled nursing facilit* | Expanders - Apply equivalent subjects  Search modes - Boolean/Phrase | 19682 |
| 6 | S3 AND S4 AND S5 | Expanders - Apply equivalent subjects  Search modes - Boolean/Phrase | 464 |

- 1. **Search B**

| # | Query | Limiters/Expanders | Results |
| --- | --- | --- | --- |
| 1 | meal* OR mealtime OR breakfast OR lunch OR dinner OR tea OR eat* OR feed* OR feeding practices OR swallowing OR dysphagia OR nutrition | Expanders - Apply equivalent subjects  Search modes - Boolean/Phrase | 9353 |
| 2 | food intake | Expanders - Apply equivalent subjects  Search modes - Boolean/Phrase | 1218 |
| 3 | S1 OR S2 | Expanders - Apply equivalent subjects  Search modes - Boolean/Phrase | 9425 |
| 4 | policy OR policies OR evidence OR evidence based OR guideline* OR process* OR structure* OR best practice* OR govern* OR protocol* OR accreditation OR standard* | Expanders - Apply equivalent subjects  Search modes - Boolean/Phrase | 64972 |
| 5 | quality OR quality of life | Expanders - Apply equivalent subjects  Search modes - Boolean/Phrase | 21623 |
| 6 | S4 OR S5 | Expanders - Apply equivalent subjects  Search modes - Boolean/Phrase | 76581 |
| 7 | residential aged care facilit* OR aged care OR residential care OR care home* OR high level care OR long term care facilit* OR skilled nursing facilit* | Expanders - Apply equivalent subjects  Search modes - Boolean/Phrase | 19682 |
| 8 | S3 AND S6 AND S7 | Expanders - Apply equivalent subjects  Search modes - Boolean/Phrase | 807 |

1. **CINAHL**
   1. **Search A**

| # | Query | Limiters/Expanders | Results |
| --- | --- | --- | --- |
| 1 | meal* OR mealtime OR breakfast OR lunch OR dinner OR tea OR eat* OR feed* OR feeding practices OR swallowing OR dysphagia OR nutrition | Expanders - Apply equivalent subjects  Search modes - Boolean/Phrase | 306174 |
| 2 | food intake | Expanders - Apply equivalent subjects  Search modes - Boolean/Phrase | 23544 |
| 3 | S1 OR S2 | Expanders - Apply equivalent subjects  Search modes - Boolean/Phrase | 315278 |
| 4 | experience* OR attitude* OR opinion* OR view* OR feeling* OR perspective* OR perception* | Expanders - Apply equivalent subjects  Search modes - Boolean/Phrase | 1051243 |
| 5 | residential aged care facilit* OR aged care OR residential care OR care home* OR high level care OR long term care facilit* OR skilled nursing facilit* | Expanders - Apply equivalent subjects  Search modes - Boolean/Phrase | 99381 |
| 6 | S3 AND S4 AND S5 | Expanders - Apply equivalent subjects  Search modes - Boolean/Phrase | 1461 |
| 7 | S3 AND S4 AND S5 | Expanders - Apply equivalent subjects  Narrow by Language: - english  Search modes - Boolean/Phrase | 1403 |
| 8 | S3 AND S4 AND S5 | Expanders - Apply equivalent subjects  Search modes - Boolean/Phrase | 79 |

- 1. **Search B**

| # | Query | Limiters/Expanders | Results |
| --- | --- | --- | --- |
| 1 | meal* OR mealtime OR breakfast OR lunch OR dinner OR tea OR eat* OR feed* OR feeding practices OR swallowing OR dysphagia OR nutrition | Expanders - Apply equivalent subjects  Search modes - Boolean/Phrase | 306174 |
| 2 | food intake | Expanders - Apply equivalent subjects  Search modes - Boolean/Phrase | 23544 |
| 3 | S1 OR S2 | Expanders - Apply equivalent subjects  Search modes - Boolean/Phrase | 315278 |
| 4 | residential aged care facilit* OR aged care OR residential care OR care home* OR high level care OR long term care facilit* OR skilled nursing facilit* | Expanders - Apply equivalent subjects  Search modes - Boolean/Phrase | 99381 |
| 5 | ""policy OR policies OR evidence OR evidence based OR guideline* OR process* OR structure* OR best practice* OR govern* OR protocol* OR accreditation OR standard*"" | Expanders - Apply equivalent subjects  Search modes - Boolean/Phrase | 1761147 |
| 6 | (""policy OR policies OR evidence OR evidence based OR guideline* OR process* OR structure* OR best practice* OR govern* OR protocol* OR accreditation OR standard*"") AND (S3 AND S4 AND S5) | Expanders - Apply equivalent subjects  Search modes - Boolean/Phrase | 2223 |
| 7 | (""policy OR policies OR evidence OR evidence based OR guideline* OR process* OR structure* OR best practice* OR govern* OR protocol* OR accreditation OR standard*"") AND (S3 AND S4 AND S5) | Expanders - Apply equivalent subjects  Narrow by Language: - english  Search modes - Boolean/Phrase | 2157 |
| 8 | (""policy OR policies OR evidence OR evidence based OR guideline* OR process* OR structure* OR best practice* OR govern* OR protocol* OR accreditation OR standard*"") AND (S3 AND S4 AND S5) | Expanders - Apply equivalent subjects  Narrow by SubjectGeographic: - australia & new zealand  Narrow by Language: - english  Search modes - Boolean/Phrase | 119 |

1. **ERIC**
   1. **Search A** (with limits applied)

**((meal* OR mealtime OR breakfast OR lunch OR dinner OR tea OR eat* OR feed* OR (feeding practices) OR swallowing OR dysphagia OR nutrition OR (food intake)) AND (experience* OR attitude* OR opinion* OR view* OR feeling* OR perspective* OR perception*) AND ((residential aged care facilit*) OR (aged care) OR (residential care) OR (care home*) OR (high level care) OR (long term care facilit*) OR (skilled nursing facilit*)))** AND (la.exact("ENG") NOT subt.exact("foreign countries" OR "child health" OR "early childhood education" OR "child development" OR "student attitudes" OR "infants" OR "learning activities" OR "teaching methods" OR "child caregivers" OR "children" OR "adolescents" OR "child care" OR "parent attitudes" OR "young children" OR "parent child relationship" OR "child rearing" OR "preschool children" OR "day care" OR "teacher attitudes" OR "preschool education" OR "academic achievement" OR "poverty" OR "secondary education" OR "elementary secondary education" OR "parent education" OR "postsecondary education" OR "parent participation" OR "toddlers" OR "child welfare" OR "college students" OR "elementary school students" OR "higher education" OR "student evaluation" OR "child abuse" OR "high schools" OR "mothers" OR "educational attainment" OR "parents" OR "early intervention" OR "educational quality" OR "family day care" OR "parent role" OR "day care centers" OR "early parenthood" OR "educational environment"))

- 1. **Search B** (with limits applied)

**((meal* OR mealtime OR breakfast OR lunch OR dinner OR tea OR eat* OR feed* OR (feeding practices) OR swallowing OR dysphagia OR nutrition OR (food intake)) AND (policy OR policies OR evidence OR (evidence based) OR guideline* OR process* OR structure* OR (best practice*) OR govern* OR protocol* OR accreditation OR standard* OR quality OR (quality of life)) AND ((residential aged care facilit*) OR (aged care) OR (residential care) OR (care home*) OR (high level care) OR (long term care facilit*) OR (skilled nursing facilit*)))** AND (la.exact("ENG") NOT subt.exact("early childhood education" OR "child health" OR "foreign countries" OR "children" OR "child development" OR "child caregivers" OR "poverty" OR "day care" OR "child welfare" OR "infants" OR "child care" OR "adolescents" OR "preschool education" OR "elementary secondary education" OR "family day care" OR "learning activities" OR "child abuse" OR "young children" OR "early parenthood" OR "postsecondary education" OR "academic achievement" OR "day care centers" OR "secondary education" OR "child neglect" OR "child rearing" OR "child safety" OR "parent participation" OR "preschool children" OR "toddlers" OR "parent child relationship" OR "foster care" OR "homeless people" OR "teaching methods" OR "child care centers" OR "parent education"))

1. **Medline, 5. Emcare and 6. PsychInfo**
   1. **Search A**

| # | Query |
| --- | --- |
| 1 | Meals/ |
| 2 | mealtime.mp. [mp=title, abstract, original title, name of substance word, subject heading word, floating sub-heading word, keyword heading word, organism supplementary concept word, protocol supplementary concept word, rare disease supplementary concept word, unique identifier, synonyms] |
| 3 | Eating/ |
| 4 | swallowing.mp. or Deglutition/ |
| 5 | dysphagia.mp. or Deglutition Disorders/ |
| 6 | (experience* or attitude* or opinion* or view* or feeling* or perspective*).mp. or perception*.tw,ab. [mp=title, abstract, original title, name of substance word, subject heading word, floating sub-heading word, keyword heading word, organism supplementary concept word, protocol supplementary concept word, rare disease supplementary concept word, unique identifier, synonyms] |
| 7 | Homes for the Aged/ or Nursing Homes/ or Aged/ or residential care.mp. or Long-Term Care/ or Residential Facilities/ |
| 8 | 1 or 2 or 3 or 4 or 5 |
| 9 | 6 and 7 and 8 |
| 10 | 2 and 6 and 7 |

- 1. **Search B**

| # | Query |
| --- | --- |
| 1 | Meals/ |
| 2 | mealtime.mp. [mp=title, abstract, original title, name of substance word, subject heading word, floating sub-heading word, keyword heading word, organism supplementary concept word, protocol supplementary concept word, rare disease supplementary concept word, unique identifier, synonyms] |
| 3 | Eating/ |
| 4 | swallowing.mp. or Deglutition/ |
| 5 | dysphagia.mp. or Deglutition Disorders/ |
| 6 | Homes for the Aged/ or Nursing Homes/ or Aged/ or residential care.mp. or Long-Term Care/ or Residential Facilities/ |
| 7 | 1 or 2 or 3 or 4 or 5 |
| 8 | Evidence-Based Practice/ |
| 9 | policy/ |
| 10 | guideline/ |
| 11 | 8 or 9 or 10 |
| 12 | 1. and 7 and 11 |

1. **Scopus**
   1. **Search A** (Limits: Australia)

( TITLE-ABS-KEY ( meal*  OR  mealtime  OR  breakfast  OR  lunch  OR  dinner  OR  tea  OR  eat*  OR  feed*  OR  "feeding practices"  OR  swallowing  OR  dysphagia  OR  nutrition  OR  "food intake" ) )  AND  ( TITLE-ABS-KEY ( experience*  OR  attitude*  OR  opinion*  OR  view*  OR  feeling*  OR  perspective*  OR  perception* ) )  AND  ( TITLE-ABS-KEY ( "residential aged care facilit*"  OR  "aged care"  OR  "residential care"  OR  "care home*"  OR  "high level care"  OR  "long term care facilit*"  OR  "skilled nursing facilit*" ) )

- 1. **Search B** (Limits: Australia)

( TITLE-ABS-KEY ( meal*  OR  mealtime  OR  breakfast  OR  lunch  OR  dinner  OR  tea  OR  eat*  OR  feed*  OR  "feeding practices"  OR  swallowing  OR  dysphagia  OR  nutrition  OR  "food intake" ) )  AND  ( TITLE-ABS-KEY ( "residential aged care facilit*"  OR  "aged care"  OR  "residential care"  OR  "care home*"  OR  "high level care"  OR  "long term care facilit*"  OR  "skilled nursing facilit*" ) )  AND  ( TITLE-ABS-KEY ( policy  OR  policies  OR  evidence  OR  "evidence based"  OR  guideline*  OR  process*  OR  structure*  OR  "best practice*"  OR  govern*  OR  protocol*  OR  accreditation  OR  standard*  OR  quality  OR  "quality of life" ) )

1. **Science Direct**
   1. **Search A**

mealtime AND experience AND 'aged care'

- 1. **Search B**

mealtime AND policies AND guidelines AND evidence AND 'aged care' AND elderly

1. **PubMed**
   1. **Search A**

("mealtime") AND ("residential aged care")

- 1. **Search B**

("residential aged care") AND (("policy") OR ("evidence") OR ("guidelines")) AND ("mealtime")

1. **Google Scholar**
   1. **Search A**

Mealtime experience residential aged care

- 1. **Search B**

Mealtime policies residential aged care

1. **Google**
   1. **Search A**

Australian aged care policies

- 1. **Search B**

Australian Royal Commission into Aged Care Quality and Safety
