# Supplementary material for: How are the mealtime experiences of people in residential aged care facilities informed by policy and best practice guidelines? A scoping review
Source: BMC Geriatr. 2022 Sep 9;22:737. doi: 10.1186/s12877-022-03340-9 (PMC9463738; doi:10.1186/s12877-022-03340-9)
Supplement: Supplementary file 3 — Additional file 3. Number of Results from Database and Engine Search. [file 12877_2022_3340_MOESM3_ESM.docx]

**Appendix C: Number of Results from Database and Engine Search**

| **Database Search** | | | |
| --- | --- | --- | --- |
|  | **Search A** | **Search B** | **Combined (Duplicates Removed)** |
| **Ageline** | 464 | 807 | 998 |
| **CINAHL** | 79 | 119 | 131 |
| **ERIC** | 59 | 130 | 155 |
| **Medline** | 120 | 12 | 132 |
| **Emcare** | 80 | 107 | 186 |
| **PsychInfo** | 27 | 0 | 27 |
| **PubMed** | 9 | 4 | 9 |
| **Science Direct** | 375 | 207 | 525 |
| **Scopus** | 122 | 205 | 235 |
| **Search Engines** | | | |
|  | **Search A** | **Search B** | **Combined (Duplicates Removed)** |
| **Google Scholar** | 200 | 200 | 290 |
|  | **Search A** | **Search B** | **Combined (Duplicates Removed)** |
| **Google (Policy Search)** | 28 | 23 | 51 |
